# Design and application of circular RNAs with protein-sponge function

**DOI:** 10.1093/nar/gkaa1085

**Published:** 2020-11-24

**Authors:** Silke Schreiner, Anna Didio, Lee-Hsueh Hung, Albrecht Bindereif

**Affiliations:** Institute of Biochemistry, Justus Liebig University of Giessen, 35392 Giessen, Germany; Institute of Biochemistry, Justus Liebig University of Giessen, 35392 Giessen, Germany; Institute of Biochemistry, Justus Liebig University of Giessen, 35392 Giessen, Germany; Institute of Biochemistry, Justus Liebig University of Giessen, 35392 Giessen, Germany

## Abstract

Circular RNAs (circRNAs) are a class of noncoding RNAs, generated from pre-mRNAs by circular splicing of exons and functionally largely uncharacterized. Here we report on the design, expression, and characterization of artificial circRNAs that act as protein sponges, specifically binding and functionally inactivating hnRNP (heterogeneous nuclear ribonucleoprotein) L. HnRNP L regulates alternative splicing, depending on short CA-rich RNA elements. We demonstrate that designer hnRNP L-sponge circRNAs with CA-repeat or CA-rich sequence clusters can efficiently and specifically modulate splicing-regulatory networks in mammalian cells, including alternative splicing patterns and the cellular distribution of a splicing factor. This new strategy can in principle be applied to any RNA-binding protein, opening up new therapeutic strategies in molecular medicine.

## INTRODUCTION

Circular RNAs (circRNAs) exist in all eukaryotes investigated so far and have been known more than four decades, starting with the plant-pathogenic viroid RNAs (1), followed by other singular examples (e.g. references 2–8). Only around 2012, circRNAs were rediscovered as a large class of noncoding RNAs, based on deep sequencing and bioinformatic screening for circRNA-specific splice junctions (‘back-splice’; references 9–11). This most common type of circRNAs consists of one or several adjacent exons derived from pre-mRNAs (reviewed by references 12–14). Biogenesis of exonic circRNAs relies on a kind of alternative splicing, as our detailed mutational analysis indicated (15).

Functionally, however, circRNAs remain largely unexplored until today, except for a miRNA sponge function, experimentally validated only for few cases (16,17). As shown for a natural miRNA sponge, circRNAs are embedded in regulatory networks of other noncoding RNAs and mRNA (18,19). Several other, hypothetical roles have been proposed for circRNAs, for example protein complex assembly, antisense activity, and protein sponging (20). CircRNAs exist in the cellular context as RNA-protein complexes, and there is clear specificity of certain RNA-binding proteins for circRNA subgroups (21). Based on their unusually high stability, circRNAs provide an attractive basis for constructing designer circRNAs for biotechnological applications (for example, see reference 22).

To experimentally test whether circRNAs can efficiently function as protein sponges, we designed, expressed and characterized artificial circRNAs as sponges for hnRNP L. HnRNP L, a classical RNA-binding protein with four RNA-recognition motifs (RRMs), can function either as splice activator or repressor, requiring binding to short CA-repeat or CA-rich RNA elements in its target pre-mRNAs (22–29). Here, we designed short circRNAs carrying either CA-repeat elements or SELEX-derived, CA-rich high-affinity binding sites for hnRNP L; circRNAs were generated either *in vitro* by T7 transcription and RNA ligation, alternatively by an *in vitro* ribozyme-mediated processing pathway (PIE system; reference 30), or they were overexpressed in cell culture (Tornado system; reference 31). We demonstrate that different type of designer hnRNP L-sponge circRNAs with CA-repeat or CA-rich sequence clusters efficiently and specifically bind hnRNP L, regulate hnRNP L-dependent splicing networks in mammalian cells, and modulate the cellular distribution of hnRNP L. Gene-specific validation assays show a strong correlation between alternative splicing effects induced by circRNA-mediated hnRNP L sponging or classical siRNA-mediated RNA interference. In sum, our approach introduces a new kind of interference strategy, usable in principle for any specific RNA-binding protein, and opening up new therapeutic strategies.

## MATERIALS AND METHODS

### PIE and Tornado plasmid constructs, stable cell lines, oligonucleotides

#### PIE-(CA)_100_ and PIE-control

For PIE-(permuted-intron–exon) constructs, the PIE expression cassette, which relies on group I splicing-mediated *in vitro* circularization (30), was synthesized (Geneart, Invitrogen) and cloned between the HindIII and XbaI sites of pcDNA3. The resulting pcDNA3-PIE vector contains a multiple cloning site (BamHI-XhoI), in which inserts to be circularized are cloned. To generate PIE-(CA)_100_, an insert with ∼100 CA-dinucleotide repeats was produced by ligation of short CA-repeat sequences joined by BamHI–BglII linkers; the resulting circRNA (CA)_100_ is 365 nts in length; the negative control, PIE-control, contains vector sequences, producing a circRNA of 484 nts.

#### Tornado-(CA)_20_, Tornado-(CA)_100_, and Tornado–control; Tornado CA-SELEX X2 and -X4

For Tornado circRNA expression, pAV-U6+27-Tornado-Broccoli and pAV-U6+27-Tornado-F30-Broccoli vectors were used (Addgene; 31). Inserts with the (CA)_20_, (CA)_100_, control, CA-SELEX X2 and CA-SELEX X4 sequences were synthesized, or PCR-amplified from the corresponding PIE constructs, and cloned between the NotI and SacII sites of pAV-U6+27-Tornado-Broccoli, replacing the Broccoli aptamer sequence. The SELEX X2 and X4 constructs contain two or four copies, respectively, of a 20-nts CA-rich sequence, derived from our initial SELEX study and validated as a high-affinity target sequence of hnRNP L (5′-AUACAUGACACACACACGCA-3′; *K*_D_ 7.2 nM; reference 25), with each of the 20-nts sequences separated by an AUAU spacer. After transfection, circRNAs Tornado (CA)_20_ (87 nts), Tornado CA-SELEX X2 (87 nts), and Tornado-CA-SELEX X4 (149 nts), were overexpressed. As an additional control, the vector pAV-U6+27-Tornado-Broccoli, expressing a circRNA of 96 nts, was used. The Tornado-(CA)_100_, the Tornado-control, and the Tornado-CA-SELEX X4 constructs were also made with the Broccoli aptamer, by cloning into the KflI site of the pAV-U6+27-Tornado-F30-Broccoli vector, resulting in circRNAs Tornado-(CA)_100_ (284 nts, with Broccoli 410 nts), the Tornado-control (379 nts, with Broccoli 500 nts), and the Tornado-CA-SELEX X4 (149 nts, with Broccoli 277 nts).

HEK293 cell lines that stably and inducibly express circRNAs were generated, based on a genomic integration construct: The (CA)_100_ unit [see above under PIE-(CA)_100_], or the negative control sequence, were cloned between the EcoRV and SacII sites of the pcDNA3.1 (+) ZKSCAN1 MCS exon vector (Addgene; reference 32), followed by recloning of the HindIII-XhoI fragment containing the (CA)_100_ unit (or the negative control sequence) and flanking inverted repeats (32) into the pcDNA5/FRT/TO genomic integration vector (Thermo Fisher Scientific). In addition, the BGH polyadenylation signal was deleted by PCR with inverse primers (see Supplementary Table S2), resulting in the construct used for stable genomic integration, which relied on the tetracycline-inducible Flp-In™ T-Rex™ System (Thermo Fisher Scientific).

For a complete list of DNA- and RNA-oligonucleotides, see Supplementary Table S2.

### *In vitro* transcription and circularization of short circRNAs

RNAs were synthesized by *in vitro* transcription, using double-stranded DNA-oligonucleotide templates and the HiScribe™ T7 High Yield RNA Synthesis Kit (NEB), and labeling internally by incorporation of azide-modified UTP analog (5-Azido-C3-UTP, 33%; Jena Bioscience), followed by RQ1 DNase treatment (10 U per 100 μl-reaction; Promega) and Sephadex column purification (Sigma-Aldrich). For circularization, T4 RNA ligase (100 U per 100 μl-reaction; Thermo Fisher Scientific) was used, followed by biotin labeling with copper-free Click Chemistry (DBCO-PEG4-Biotin conjugate; Jena Bioscience).

### HnRNP L sponging assays: biotin pull-down and RNA immunoprecipitation (RIP)

HeLa cells were lysed in RIPA buffer [50 mM Tris–Cl pH 7.4, 150 mM NaCl, 5 mM EDTA, 1% NP-40 (v/v)]. For *in vitro* binding assays, 50 pmol of biotinylated RNA was prebound to 30 μl MyOne Streptavidin C1 Dynabeads (binding capacity ∼5000 pmol/ml packed beads; Thermo Fisher Scientific), followed by incubation with 20 μl HeLa cell lysate (corresponding to 2.2 × 10^5^ cells) and washing off unbound proteins at 300 mM KCl. Bound proteins were released and separated by 10% SDS-PAGE, followed by Western blotting with hnRNP L, GAPDH (Sigma-Aldrich), or IMP3 (Millipore) primary antibodies and peroxidase-coupled secondary antibodies (Sigma-Aldrich).

For assaying hnRNP L sponging *in vivo*, antibodies were added to pre-cleared lysate (6 μg antibody per immunoprecipitation; anti-hnRNP L and anti-Flag, Sigma-Aldrich; anti-IMP3, Millipore; 350 μl lysate, corresponding to 1.8 × 10^6^ cells), incubated overnight at 4°C, followed by addition of 30 μl (1 mg) Protein A or G Dynabeads (Thermo Fisher Scientific), and rotation for two hours at 4°C. Protein–RNA complexes were washed by increasing the stringency up to 600 mM NaCl. RNA from input and immunoprecipitated fractions was extracted by TRIzol (Ambion), followed by reverse transcription (qScript cDNA SuperMix, containing dNTPs, MgCl_2_, primers, RNase inhibitor, qScript™ reverse transcriptase, and stabilizers; Quanta) and (q)PCR with gene- and linear/circular-specific primers. The fraction of bound target RNAs was calculated for each target relative to the corresponding input fraction.

### PIE-mediated circularization *in vitro* of long circRNAs; direct RNA analysis

For PIE (permuted-intron–exon)-mediated circularization *in vitro* (30), RNA was first *in vitro* transcribed (XbaI run-off; HiScribe™ T7 High Yield RNA Synthesis Kit, New England Biolabs), followed by RQ1 DNase treatment (Promega) and purification by the Monarch RNA Cleanup Kit (New England Biolabs). Ribozyme-catalyzed circularization was induced in splicing buffer [T4 RNA ligase buffer (50 mM Tris–Cl pH 7.5, 10 mM MgCl_2_, 1 mM DTT); New England Biolabs] by the addition of GTP (final concentration of 2 mM), followed by incubation for 8 min at 55°C. Circularization efficiency was checked on the 2% E-Gel system (Thermo Fisher Scientific). To purify circRNAs further, RNA was treated with RNase R (Lucigen) and HPLC-fractionated (for details, see Supplementary Figure S2 and reference 30). The corresponding linear RNAs were obtained by *in vitro* XbaI run-off transcription without the subsequent circularization step.

RNAs were analyzed directly by electrophoresis in denaturing polyacrylamide gels (PAGE, 12% or 15%), agarose gel (1.5%), or by E-gel electrophoresis (2% or 4%; Thermo Fisher Scientific), and stained by SYBR Gold; in case of Broccoli-carrying RNAs, gels were first stained with DFHBI (Broccoli staining; Sigma-Aldrich). For RNA analysis, either low- or high-range RNA markers were used (Thermo Fisher Scientific).

### CircRNA expression: alternative splicing, cellular distribution of hnRNP L protein and circRNAs

For transfection of PIE circRNA, 5 × 10^4^ HeLa cells were seeded in 24-well plates one day before transfection, and 100 or 500 ng linear or circular RNA were transfected, using Lipofectamine™ MessengerMax™ mRNA transfection reagent (Thermo Fisher Scientific), and harvested after 24 h. RNA was isolated using TRIzol (Ambion) and RNeasy columns (Qiagen), followed by reverse transcription (qScript cDNA Synthesis Kit; Quanta) and alternative splicing assays by PCR with gene-specific primers.

For transfection of Tornado circRNA expression constructs, HeLa cells were seeded onto 10 cm plates (1  ×  10^6^ cells per plate) one day before transfection. Transfection was performed by the TurboFect reagent (Thermo Fisher Scientific). After 48–72 h, total RNA was isolated using Norgen kit (Norgen Biotek). For the time course experiment, RNA was isolated 1, 2, 3 and 4 days post-transfection. For alternative splicing assays, total RNA (1 μg) was primed by oligo (dT)_20_ and reverse-transcribed (qScript™ Flex cDNA synthesis kit, Quanta), followed by PCR assays using gene-specific primers (for primer sequences, see Supplementary Table S2). For standard agarose gel electrophoresis of RT-PCR products, DNA markers were used [GeneRuler Ladder Mix with 500 (as reference band), 400, 300, 200, and 100 bp; Thermo Fisher Scientific].

The cellular distribution of hnRNP L protein was assayed by a detergent-based protocol for cell fractionation to obtain soluble nuclear and cytoplasmic proteins (NE-PER™ Kit, Thermo Fisher Scientific), followed by Western blot analysis for hnRNP L (Sigma-Aldrich), GAPDH (Sigma-Aldrich), and hnRNP A1 (Santa Cruz Biotechnology). Proteins were quantified by densitometry, using the ImageJ software, and based on biological replicates. In addition, the nucleo-cytoplasmic distribution of circRNAs and linear RNAs was determined by RT-qPCR.

### Determination of absolute concentrations of overexpressed circRNAs and of cellular hnRNP L

To determine the absolute concentration of circRNAs (copy number per cell), total RNA was isolated from HeLa cells, using TRIzol (Ambion) after transfection of Tornado-expression plasmid DNA, or using TRIzol (Ambion) and RNeasy columns (QIAGEN) after transfection of RNAs expressed by the PIE system. Total RNA (200 ng) was reverse-transcribed by qScript reverse transcriptase (Quanta) followed by real-time PCR carried out in triplicates. As quantitative standards, we used control, (CA)_100_, CA-SELEX X4, and CA-SELEX X4/Broccoli circRNAs. 50 ng of the RNA transcript and 200 ng total RNA as competitor was reverse-transcribed (see above). Based on that, a standard curve (five 10-fold dilutions from 5 to 0.0005 ng) was derived to determine absolute quantities using the NEBiocalculator software. Real-time PCR was carried out using Luna^®^ Universal qPCR Master Mix (NEB) on an Eppendorf realplex2 thermocycler. Standard-curve *R*^2^ values were >0.99 and amplification efficiency between 90% and 100%.

To determine the corresponding concentration of hnRNP L, HeLa cell lysate was prepared as described above and analyzed by Western blotting for hnRNP L and GAPDH (see above), using 1, 2.5, and 5 μl (3.7 × 10^4^ cells/μl) on a 10% SDS-polyacrylamide gel, and comparing signals obtained with recombinant GST-hnRNP L protein (5, 10, 30 and 50 ng, based on a standard curve with BSA (Roche).

### Global analysis of alternative splicing by RNA-seq

For global analysis of sponging, 2.5 × 10^5^ HeLa cells were seeded one day before transfection with 0.5, 1 or 2.5 μg (CA)_100_ or control circRNA, using Lipofectamine™ MessengerMax™ mRNA transfection reagent (Thermo Fisher Scientific). For RNAi-knockdown, 8.8 × 10^5^ cells were reverse-transfected with RNAiMAX (Thermo Fisher Scientific), using siRNAs specific for human hnRNP L and luciferase GL2 (Sigma-Aldrich). 72 h post-transfection, total RNA was isolated, quality-controlled and depleted of ribosomal RNA (NEBNext rRNA depletion kit), followed by library preparation (NEBNext Ultra Directional RNA Library Prep Kit) and sequencing on Illumina NextSeq 500 (single-end read, 150 bp). RNA-seq data were deposited in the Sequence Read Archive (PRJNA610182) of NCBI.

Sequence reads were aligned to the human genome sequence (hg19 assembly) using STAR (33). The comprehensive gene annotation set from GENCODE Version 19 (http://www.gencodegenes.org) was applied for gene expression and splicing analyses. From each of the six sponge samples, ∼81% (81.01–82.15%) of the sequenced reads (51.37–59.91 mio) were uniquely mapped, in the two siRNA-knockdown samples, ∼77% of the sequenced reads (62.53 and 74.22 mio).

For global gene expression analysis, the read coverage of annotated protein coding genes were normalized with the number of uniquely mapped reads in each sample and with the mRNA length. Normalized read coverage from ∼9200 genes, of which at least one sample had a minimum read coverage of 16, was used to calculate the expression ratios between samples (MA plots in Figure 4D).

For predicting increased single exon skipping upon CA-sponging by circRNA, junction read counts for exon skipping and inclusion were used to calculate the ratio of skipping versus inclusion events (34). We first obtained this ratio (sk:incl) for each sample. Second, the ratios (R_) between samples with corresponding amounts of transfected (CA)_100_ and control circRNAs (0.5, 1.0 and 2.5 μg) were calculated as following:

R_0.5 = log_2_ (sk:incl_CA_100__0.5 μg) – log_2_ (sk:incl_control_0.5 μg);
R_1.0 = log_2_ (sk:incl_CA_100__1.0 μg) – log_2_ (sk:incl_control_1.0 μg);
R_2.5 = log_2_ (sk:incl_CA_100__2.5 μg) – log_2_ (sk:incl_control_2.5 μg).

Positive targets were selected based on the dose-dependent increase in skipping/inclusion effects after circRNA transfection (R_1.0 – R_0.5 > 0.75 or R_2.5 – R_0.5 > 0.75); in addition, to reduce the false positive ratio, a minimum skipping junction read counts of 16 was required in the (CA)_100_ samples. For the prediction of increased single exon inclusion upon CA-sponging by circRNA, the analogous procedure was applied for the ratio of exon inclusion versus skipping events (incl:sk).

For the siRNA-knockdown approach, corresponding ratios (*R*) were calculated between samples with hnRNP L-specific versus luciferase-control siRNA, and positive targets were predicted, if *R* values were >0.75. In addition, a minimum of 16 skipping junction read counts was required.

## RESULTS AND DISCUSSION

### Sponging hnRNP L *in vitro* by small CA-repeat circRNAs

**Figure 1.**
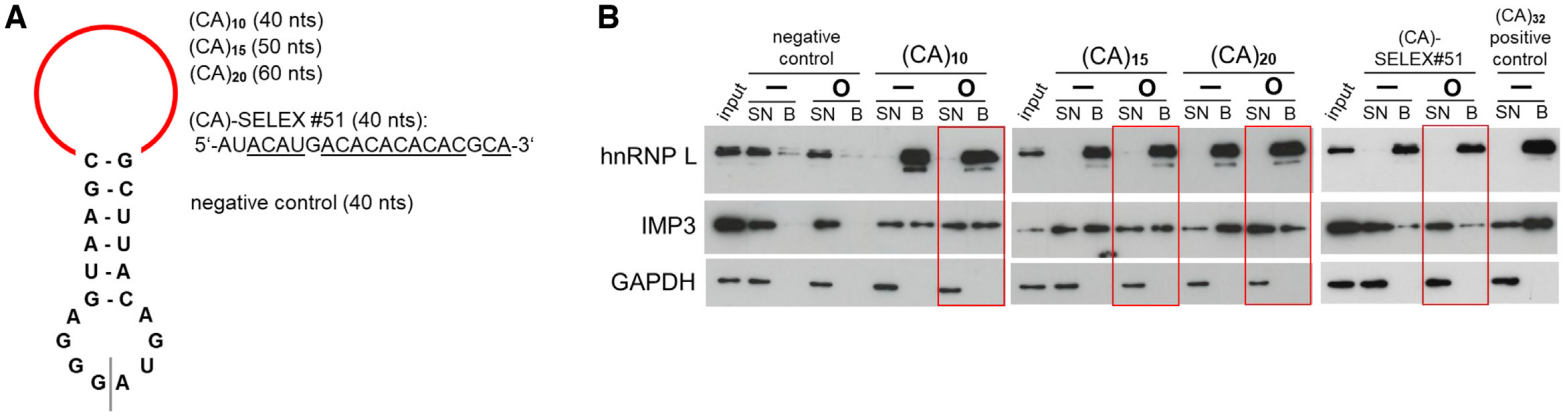
Sponging hnRNP L *in vitro* by small CA-repeat circRNAs. (**A**) Small circRNAs used for *in vitro* hnRNP L sponging were synthesized by T7-transcription and circularization, all based on the same stem-loop and differing in their upper loop sequence (in red; *in vitro* circularization site indicated by line; sizes of circRNAs in parentheses). (**B**) HnRNP L sponging *in vitro* by small circRNAs. RNAs containing 10, 15, or 20 CA-dinucleotide repeats, a CA-rich, SELEX-derived RNA sequence, as well as a negative control RNA, each in linear (−) or circular (O) configuration, were synthesized in biotinylated form. In addition, a linear (CA)_32_ RNA was used as a positive control. After incubation in HeLa cell lysate, hnRNP L binding was assayed by pulldown with streptavidin beads, followed by Western blot analysis, comparing supernatant (SN; 2.5%), bound material (B; 20%) and input (2.5%). For comparison and as specificity control, IMP3 protein binding was assayed as well. GAPDH served as an additional input control.

To produce circRNAs with specific sponge function for the RNA-binding protein hnRNP L, we initially designed short RNAs that can be efficiently synthesized by *in vitro* T7 transcription and RNA-ligase-mediated circularization (Figure 1A). Based on the known binding specificity of hnRNP L for CA-repeat and CA-rich RNA sequences, we first generated short circRNAs with a common backbone (20-nts stem-loop) and a loop comprised of 10, 15, and 20 CA-dinucleotides, resulting in circRNAs of 40, 50, and 60 nts in total, respectively: (CA)_10_, (CA)_15_, and (CA)_20_. As an alternative option, a 20-nts CA-rich sequence was used, derived from our earlier SELEX study and validated as a high-affinity target sequence of hnRNP L [(CA)-SELEX#51, called CA-SELEX in the following; 5′-AUACAUGACACACACACGCA-3′; *K*_D_ 7.2 nM; reference 25]. A linear synthetic (CA)_32_ RNA, which binds hnRNP L with high affinity (24), and a random sequence of 20 nts as a negative control (5′-CCTGCCTGTCTATTGATGTC-3′; generated by a random sequence generator tool; http://www.faculty.ucr.edu/∼mmaduro/random.htm) were synthesized as described above.

To validate hnRNP L sponging *in vitro*, four short RNAs with 10, 15, 20 CA-dinucleotides, or with the CA-SELEX hnRNP L high-affinity binding sequence, were generated with biotin incorporation, each in linear and circular configuration, as well as the negative control circRNA and a linear positive control RNA, (CA)_32_. After incubation in HeLa cell lysate, hnRNP L binding was assayed by pulldown with streptavidin beads, followed by Western blot analysis for bound hnRNP L, comparing supernatant (2.5%), bound material (20%) and input (2.5%). In addition, IMP3 binding was tested, to stringently assess specificity, since IMP3 protein recognizes an array of RNA binding sites containing also CA-rich elements (35); finally, GAPDH served as input and negative control (Figure 1B). We conclude from these *in vitro* binding assays that with each of the four high-affinity binding sequences, both in circular and linear form, as well as with the linear (CA)_32_ RNA, hnRNP L can be quantitatively bound, in contrast to the negative control RNA. Note that IMP3 also binds the short CA-repeat RNAs, although at lower efficiency than hnRNP L; in contrast, the SELEX-derived sequence is quantitatively bound by hnRNP L, but only at background levels by IMP3, indicating higher selectivity of the SELEX sequence. For assays of hnRNP L sponging *in vivo*, see below.

### Large CA-repeat circRNAs: *in vivo* hnRNP L sponging in stable cell line, ribozyme-mediated synthesis and purification

**Figure 2.**
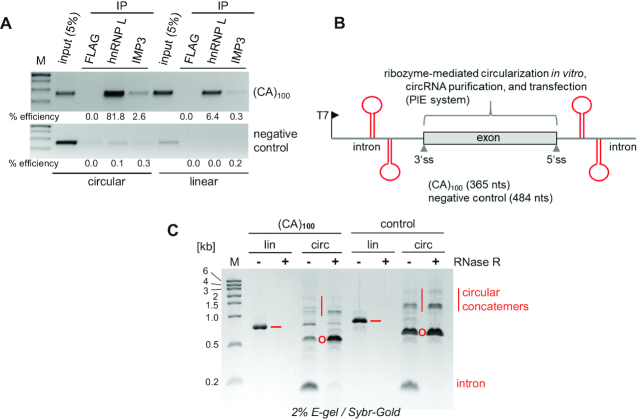
Large CA-repeat circRNAs: *in vivo* hnRNP L sponging in stable cell line, ribozyme-mediated synthesis and purification. (**A**) HnRNP L sponging *in vivo* by (CA)_100_ circRNA. Lysates were prepared from HEK293 cell lines stably expressing (CA)_100_ circRNA (top panel) or a negative control RNA (bottom panel), followed by immunoprecipitation (IP) with anti-hnRNP L, or as controls, with anti-IMP3 or -FLAG antibodies. For comparison, 5% of input lysates was applied. Immunoprecipitated circRNAs and linear precursors were detected by RT-PCR and circular-junction- and linear-precursor-specific primers. IP efficiencies are indicated below the respective lanes. *M*, DNA markers. (**B**, **C**) Expression constructs for synthesis of large circRNAs, based on the PIE self-splicing system (reference 30; sizes of circRNAs in parentheses). RNAs containing ∼100 CA-dinucleotides [(CA)_100_], or a negative control sequence, were T7-transcribed (lin), followed by *in vitro* PIE-mediated processing to circular RNA (circ), RNase R digestion (−/+), and HPLC purification (for details, see Supplementary Figure S2). Linear precursor (−), circRNA (O), released linear intron and circular concatemers are marked. RNA was analyzed by E-gel electrophoresis and visualized by SYBR Gold. *M*, RNA markers (sizes in kb).

We next focussed on longer circRNAs with more binding sites for hnRNP L. To assay hnRNP L sponging *in vivo*, we generated HEK293 cell lines, based on the Flp-In™ T-Rex™ system, that stably express -after tetracycline induction- a long circRNA, comprised of ∼100 CA-dinucleotides, (CA)_100_, or a negative control circRNA (Figure 2A). The (CA)_100_ sequence was genomically integrated, within the sequence context of two flanking inverted repeats, and is expressed from the strong CMV promoter, into which two copies of the tet operator sequence are inserted. Lysates were prepared, followed by immunoprecipitation with anti-hnRNP L, or, as specificity controls, with anti-IMP3 or anti-FLAG antibodies. Immunoprecipitated RNAs were detected by RT-PCR with primer pairs specific for the designer circRNAs, or, for comparison, for the linear precursor RNAs. We conclude that the (CA)_100_ circRNA binds hnRNP L *in vivo* with high specificity and efficiency. In contrast, the IMP3 protein, another multidomain RNA-binding protein, which recognizes also certain CA-rich sequences (35), binds only at comparatively very low efficiency (based on RT-qPCR, anti-hnRNP L: 81.8%, anti-IMP3: 2.6% efficiency). Linear (CA)_100_ precursor RNAs are detectable, and bound by hnRNP L at only 6.4% efficiency, by IMP3 at 0.3%. Why hnRNP L interacts less efficiently with the linear (CA)_100_ precursor (6.4%) than with the processed circRNA (81.8%), may be related by the nuclear localization and transient nature of the precursor. The negative control RNA bound hnRNP L and IMP3 at very low to insignificant levels (efficiencies below 0.3%). High specificity of hnRNP L binding to (CA)_100_ circRNA *in vivo* was further confirmed by direct mass-spectrometric analysis of proteins interacting *in vitro* with biotinylated (CA)_100_ circRNA in HeLa nuclear extract (Supplementary Figure S1).

To demonstrate functionality of long and biochemically characterized designer circRNAs as hnRNP L sponges, we made use of the PIE (permutated exon-intron) system. This had recently been developed as a very efficient *in vitro* expression platform for long circRNAs, relying on *in vitro* T7 transcription and circular processing through ribozyme-mediated group I splicing (30) (Figure 2B). Specifically, we expressed a (CA)_100_ circRNA (365 nts in total), as well as a negative control circRNA (484 nts), which were synthesized in both linear and circular configuration. The linear version was produced by T7 transcription, omitting the circularization step. Both circRNAs were further enriched by RNase R treatment (which digests linear RNAs in the reaction), and both circRNAs and linear RNA transcripts were finally purified by HPLC (Figure 2C and Supplementary Figure S2).

### Shifting the nuclear-cytoplasmic distribution of hnRNP L by transfected large CA-repeat circRNAs

**Figure 3.**
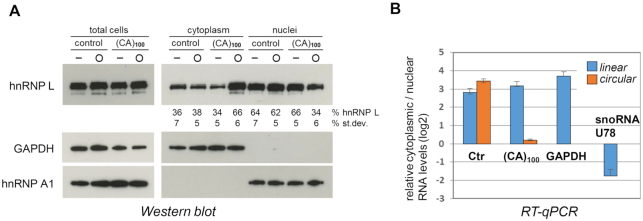
Shifting the nuclear-cytoplasmic distribution of hnRNP L by transfected large CA-repeat circRNAs. (**A**, **B**) Large hnRNP L-sponge circRNA shifts nuclear-cytoplasmic distribution of hnRNP L. (CA)_100_ RNA and control RNA, each in linear or circular configuration (−, O), were transfected in HeLa cells, followed by cell fractionation after 24 h. Equivalent lysate amounts of total cells, cytoplasmic and nuclear fractions were analyzed by Western blotting for hnRNP L, GAPDH, and hnRNP A1. The distribution of hnRNP L between nuclear and cytoplasmic fractions was quantitated, based on Western signals (mean values and standard deviations given below the respective lanes; *n* = 3; panel **A**). In addition, the nuclear-cytoplasmic distribution of control and (CA)_100_ circRNA (orange), as well as their linear precursors (blue), was quantitated by RT-qPCR, using GAPDH mRNA and U78 snoRNA as respective markers for the cytoplasmic and nuclear fractions (panel **B**).

Since hnRNP L -as a shuttling protein- is distributed between nucleus and cytoplasm, with predominantly nuclear localization (23,36), we next assayed for an effect of our long CA-repeat sponge circRNA on hnRNP L’s nuclear-cytoplasmic distribution (Figure 3A). Twenty-four hours after transfection of (CA)_100_ RNA and control RNA, each in linear or circular configuration, HeLa cells were fractionated, and equivalent lysate amounts of total cells, cytoplasmic and nuclear fractions were analyzed by Western blotting for hnRNP L, and, as controls and for normalization, for GAPDH and hnRNP A1. Based on the Western signals, in the control circRNA transfection the cytoplasmic:nuclear ratio of hnRNP L is 38:62, similar as in the transfection of linear RNAs (around 35:65); in contrast, after (CA)_100_ circRNA transfection this ratio dramatically shifts to 66:34.

We conclude that the (CA)_100_ circRNA results in a translocation of hnRNP L from the nucleus to the cytoplasm. This strong effect is specific for the (CA)_100_ circRNA and interestingly, for the circular configuration thereof. At the same time, total steady-state levels of hnRNP L protein did not significantly change. The circular-specific effect may be related to differential stabilities of circular versus linear forms, most likely also differing between nuclear and cytoplasmic residence. In any case, this relocalization of hnRNP L may open up new ways for disease therapy, since protein localization of RNA-binding proteins often changes in pathological settings and can cause disease.

Note that the cellular levels of these transfected designer circRNAs were very high, reaching 10^6^ to 10^7^ circRNA copies per cell (as quantitated by RT-qPCR; see Supplementary Figure S3A), which is in the same order of magnitude as the copy number of hnRNP L [∼10^6^ molecules per cell, estimated on the basis of Western blotting of HeLa cell lysate and using recombinant hnRNP L protein as a standard; see Supplementary Figure S3B]. Therefore, this explains the successful competition of overexpressed circRNA sponges for the abundant hnRNP L protein.

In parallel, we determined the cellular distribution of the transfected circRNAs, based on quantitative RT-PCR (Figure 3B). Whereas both linear and circular control RNAs as well as linear (CA)_100_ RNA were predominantly cytoplasmic (to ∼90%), (CA)_100_ circRNA was equally distributed between nuclear and cytoplasmic fractions, most likely reflecting the high-affinity binding of nuclear hnRNP L to this circRNA.

### Transfected large hnRNP L-sponge circRNA, (CA)_100_, regulates alternative splicing *in vivo*: principle, global target analysis, validation

**Figure 4.**
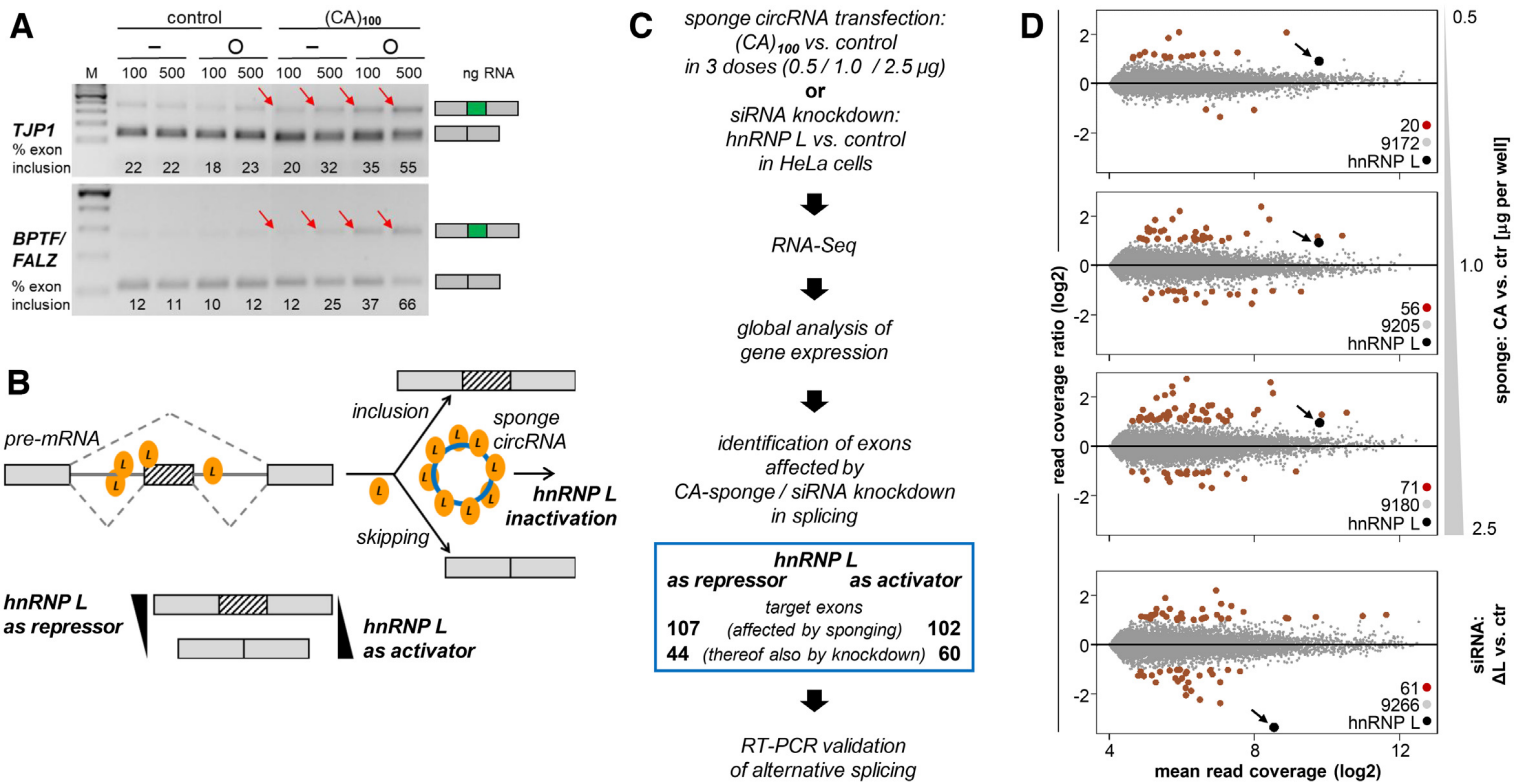
Transfected large hnRNP L-sponge circRNA, (CA)_100_, regulates alternative splicing *in vivo*: principle and global target analysis. (**A**) Alternative splicing regulation of two known hnRNP L target genes. (CA)_100_ hnRNP L-sponge and control RNAs, either in linear or circular configuration (−/O), were synthesized by the PIE-system (see Figure 2) and transfected in HeLa cells (100 or 500 ng per transfection). After 24 hr, alternative splicing was assayed by RT-PCR for two known hnRNP L targets, *TJP1* and *BPTF/FALZ*, where hnRNP L functions as a splice repressor. The two RT-PCR products indicate exon inclusion (red arrows) and skipping, respectively; quantitation of exon inclusion (in %) is indicated in the respective lanes. *M*, DNA markers. (**B**) General concept of alternative splicing modulation by CA-repeat circRNA sponges. HnRNP L regulates exon skipping and inclusion, acting either as splicing activator or repressor. These splicing decisions can be modulated by a CA-sponge circRNA, which inactivates hnRNP L by sponging, resulting in a shift in the ratio of splice isoforms (skipping / inclusion), depending on whether hnRNP L acts as a repressor or activator. (**C**) Global analysis of gene expression and alternative splicing after circRNA-based hnRNP L sponging: flowchart of analysis. (**D**) Global gene expression changes, comparing hnRNP L-sponging (CA- vs. control-circRNAs transfected in three doses: 0.5, 1.0 and 2.5 μg per sample) and hnRNP L knockdown (hnRNP L- versus control-siRNA), shown as MA plots. Mean read coverages are plotted on the X-axis, and read coverage ratios on the Y-axis. Significantly up- and down-regulated genes are plotted in red, unaffected genes in gray, and the hnRNP L gene indicated by arrow.

Next we assayed the functionality of long designer circRNAs that act as hnRNP L sponges, focussing on the established role of hnRNP L as a specific splicing regulator. (CA)_100_ hnRNP L-sponge or control RNAs, either in linear or circular configuration (lin/circ), were transfected in HeLa cells (100 or 500 ng per transfection; Figure 4A). After 24 h, alternative splicing was assayed by RT-PCR for two known hnRNP L targets, *TJP1* and *BPTF* (also called *FALZ*), where hnRNP L functions as repressor of an alternatively spliced exon (26). The two RT-PCR products indicate exon inclusion (red arrows) and skipping, respectively, and quantitation of exon inclusion (in %) is indicated in the respective lanes.

We conclude that the CA-repeat circRNA reproducibly and strongly increased exon inclusion, up to 55% (*TJP1*) and 66% (*BPTF/FALZ*). These effects were clearly specific for the (CA)-repeat sponge RNA (compare with control transfections), dosis-dependent (compare 100 and 500 ng RNA), and much more pronounced for the circRNA than for corresponding quantities of linear RNA. The extent of alternative splicing modulation observed here after CA-sponge circRNA transfection was at least comparable with the effects initially found after siRNA-mediated knockdown of hnRNP L expression (26).

We further extended this alternative splicing analysis to a genomewide level, focussing on exon skipping and inclusion, the most abundant type of alternative splicing. Since hnRNP L can act either as activator or repressor, the ratio of exon inclusion versus skipping may shift accordingly (for a schematic of this principle, see Figure 4B).

HeLa cells were transfected for one day with the purified (CA)_100_ sponge circRNA, in parallel with a control circRNA. In addition, we performed classical siRNA-based hnRNP L knockdown assays, to directly compare RNAi silencing and sponging effects by RNA-seq (Figure 4C; for Western blot analysis of hnRNP L knockdown, see Supplementary Figure S4A). We first compared gene expression between CA-sponge versus control-circRNA transfections as well as betweeen hnRNP L- versus control-knockdown samples (Figure 4D). The number of significantly up- and down-regulated genes (log_2_ ratio ≥ 1 or ≤ –1) increased dose-dependently in the circRNA-sponge transfections: 20, 56 and 71 genes in the samples with 0.5, 1.0 and 2.5 μg doses, respectively. As expected, the expression of hnRNP L mRNA was strongly reduced by siRNA-knockdown (log_2_ ratio: –3.35), while it was slightly up-regulated (log_2_ ratio of 0.94, 0.96 and 0.98 in the circRNA-sponge samples at respective 0.5, 1.0 and 2.5 μg doses), reflecting the efficient autoregulation of hnRNP L (27).

**Figure 5.**
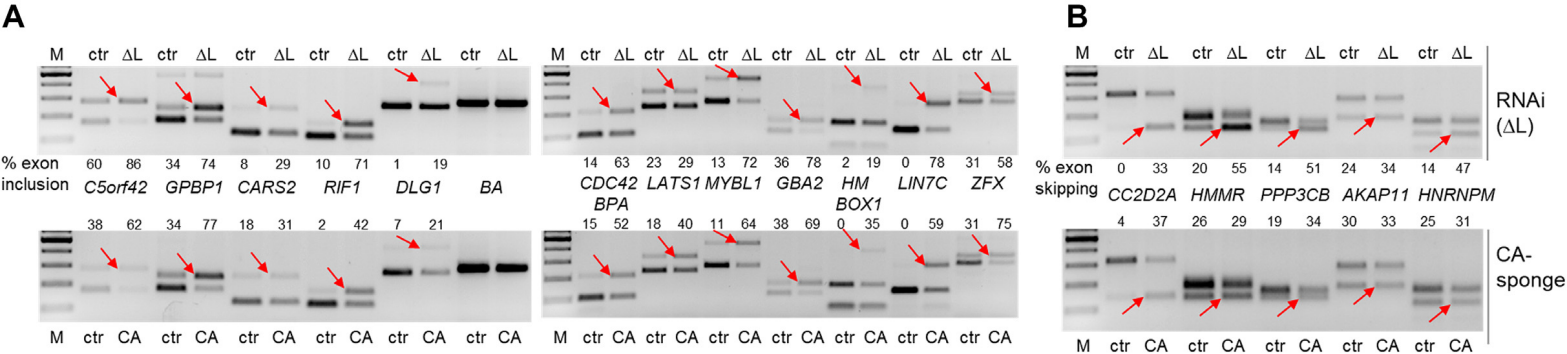
Validation of alternative splicing effects. (**A**, **B**) RNAi-knockdown (top panels: control, ctr, versus L knockdown, ΔL), and CA-sponge effects (bottom panels: transfection of control, ctr, versus CA-sponge circRNA, CA) were tested by RT-PCR and directly compared with each other (quantitation of exon inclusion in % indicated in the respective lanes). Exon inclusion versus skipping was monitored for a total of 17 predicted target exons, where hnRNP L acts as repressor (panel **A**) or activator (panel **B**). The two RT-PCR products (red arrows) indicate exon inclusion and skipping, respectively [gene names in the middle; β-actin (BA) as an unaffected control]. *M*, DNA markers.

For stringent prediction and selection of CA-sponge-specific targets of alternative splicing, we analyzed dosis-dependent changes after transfection with different quantities of circRNA [0.5, 1 or 2.5 μg (CA)_100_ circRNA; for details of data analysis, see Materials and Methods]. As a result, we were able to predict target exons that responded in their inclusion or skipping pattern to the CA-sponge circRNA (Figure 4C): 107 exons showed a significant increase of exon inclusion after CA-sponge expression, of which 44 (41%) responded in the same manner to RNAi knockdown (hnRNP L as splicing repressor). On the other hand, 102 exons showed a significant increase of exon skipping after CA-sponge expression, of which 60 (59%) were also RNAi-knockdown-responsive (hnRNP L as splicing activator). For a list of all predicted target exons, see Supplementary Tables S1A and B. Validations by semi-quantitative RT-PCR assays of a subset of these predicted targets clearly confirmed our analysis, including both activator and repressor examples of hnRNP L target exons (Figure 5A and B, respectively). Finally, the high correlation of circRNA-sponge and RNAi-induced effects on exon-specific splicing modulation (41% and 59%) strongly indicates that circRNA-based sponging efficiently inactivates hnRNP L protein.

### Overexpression of sponge circRNAs: hnRNP L binding and alternative splicing modulation

**Figure 6.**
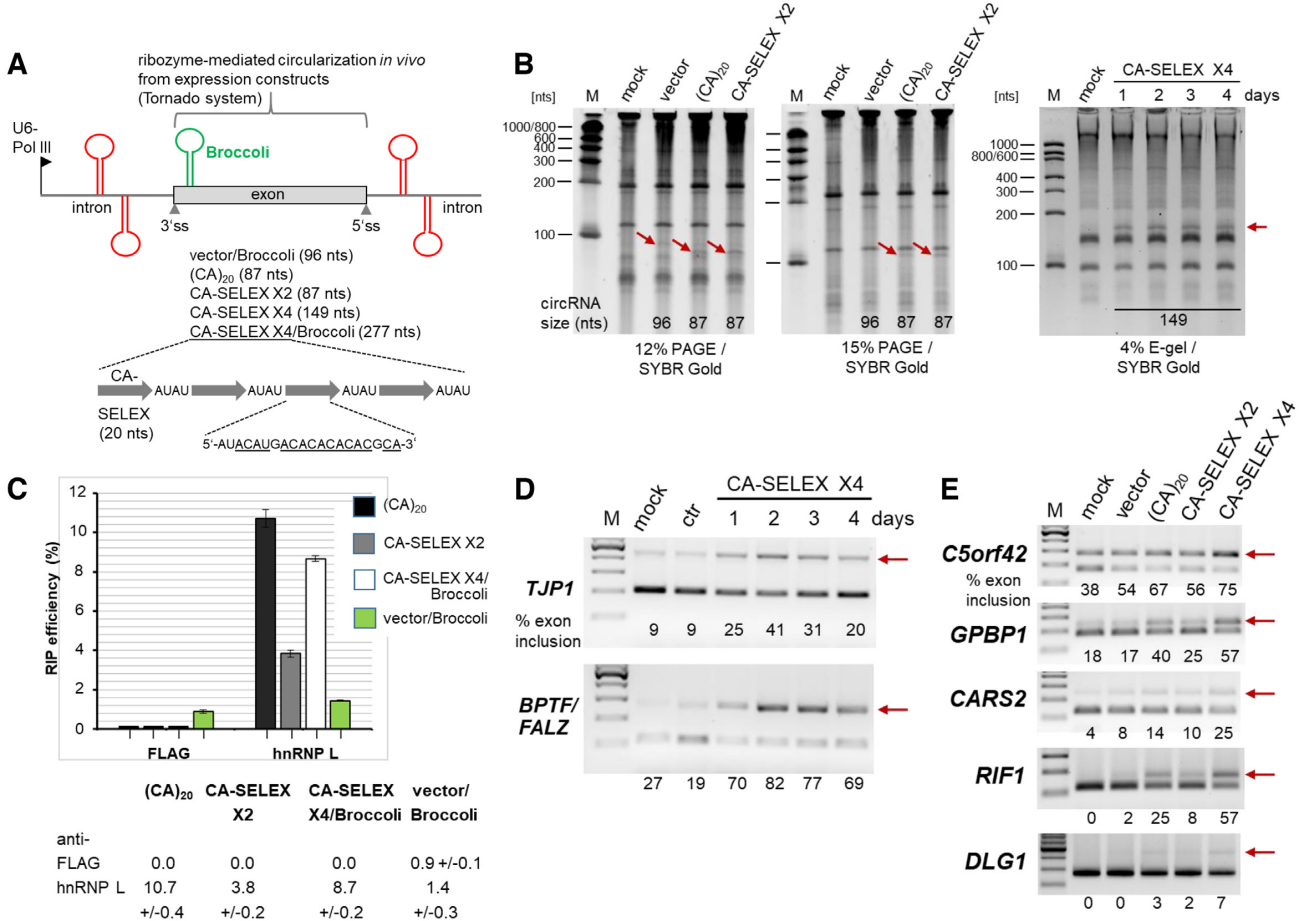
Overexpression of sponge circRNAs: hnRNP L binding and alternative splicing modulation. (**A**) CircRNA overexpression constructs, based on the Tornado self-splicing system and transient transfection (reference 31; sizes of circRNAs given in parentheses, as well as the schematic structure of CA-SELEX X4 construct). (**B**) Direct RNA analysis of circRNA overexpression in HeLa cells. Following transfection of (CA)_20_, CA-SELEX X2, and CA-SELEX X4 constructs, total RNA prepared after two days was analyzed by denaturing PAGE (left and middle panels; 3 μg each) or E-gel electrophoresis (one- to four-day time course of expression for CA-SELEX X4; 4 μg each), and visualized by SYBR Gold (mock- and Tornado-vector/Broccoli transfections as controls). *M*, RNA markers (sizes in nts). The arrows mark overexpressed circRNAs (sizes in nts below). (**C**) HnRNP L binding by overexpressed CA-sponge circRNAs. Efficiencies of anti-hnRNP L RNA immunoprecipitation (RIP) were determined after a two-day transfection of (CA)_20_, CA-SELEX X2 and CA-SELEX X4/Broccoli constructs in HeLa cells (Tornado-vector as control), based on RT-qPCR (% of input, with anti-FLAG as negative control; *n* = 3). (**D**, **E**) Overexpressed CA-sponge circRNAs modulate alternative splicing. CA-SELEX X4 circRNA was overexpressed for one to four days in HeLa cells, followed by RT-PCR-based analysis of alternative splicing. Percentages of exon inclusion (red arrows) are given below the lanes. Mock-transfected (mock), Tornado-control (ctr)- or Tornado-vector/Broccoli-transfected cells (after two days) served as controls. The sponge effects of CA-SELEX X4 circRNA, including the time dependence over four days was analyzed for two known hnRNP L targets, *TJP1* and *BPTF/FALZ* (panel **D**). Similarly, alternative splicing was assayed for five additional hnRNP L targets, comparing the sponge effects of (CA)_20_, CA-SELEX X2 and CA-SELEX X4 circRNAs (panel **E**). *M*, DNA markers.

Alternatively to circRNA transfection, we established a highly efficient overexpression system for designer hnRNP L sponge circRNAs, containing CA-repeat or CA-rich sequences. Overexpression is based on the so-called Tornado system introduced by Litke and Jaffrey (31), which relies on transient transfection of an RNA-polymerase III-driven self-cleaving expression cassette, combined with circularization by the RtcB tRNA ligase (Figure 6A). We expressed circRNAs with a short CA-repeat unit, (CA)_20_ (87 nts), as well as two (87 nts) or four copies (149 nts) of the SELEX-derived hnRNP L high–affinity RNA motif (25) used already in the *in vitro* binding assays described above (see Figure 1). Overexpression after transfection of these Tornado-based constructs was unusually high, compared with endogenous circRNAs, as all three short circRNAs were detectable by direct RNA analysis and visualization by SYBR Gold (Figure 6B; for an absolute quantitation of the Tornado-overexpressed circRNAs, see Supplementary Figure S3A). The altered mobility relative to linear RNA markers directly proved the circular configuration of the expressed (CA)_20_ and CA-SELEX X2 circRNAs (for additional evidence based on Northern blot analysis and a circular junction-specific probe, see Supplementary Figure S4B). As shown here for the CA-SELEX X4 circRNA, overexpression remained at these high levels for at least 4 days post-transfection.

Both the short (CA)_20_ circRNA as well as circRNAs with two and four copies of the SELEX-based high-affinity motif bound hnRNP L efficiently and specifically, when overexpressed in HeLa cells (Figure 6C), with efficiencies up to 10.7% in RT-qPCR-based RNA-immunoprecipitation assays. Note that due to the strong overexpression of these circRNAs, efficiencies are most likely limited by the available hnRNP L protein.

Finally, alternative splicing modulation was tested, first using again two known target genes, where hnRNP L functions as a repressor (*TJP1* and *BPTF/FALZ*), monitoring exon inclusion between one to four days post-transfection (Figure 6D). In addition, five other examples were assayed, where hnRNP L represses exon inclusion (*C5orf42*, *GPBP1*, *CARS2*, *RIF1*, *DLG1*; Figure 6E), and which had been tested above after transfection of (CA)_100_ circRNA sponge (see above and Figure 5A). We observed strong alternative-splicing effects after expression of each of the three hnRNP L sponge circRNAs (Figure 6E), ranking in strength consistently for each of the five target exons in this order: CA-SELEX X2, (CA)_20_, CA-SELEX X4 circRNA.

In addition to these short CA-repeat and CA-rich circRNAs, we also tested the longer (CA)_100_ circRNA, which we had characterized after circRNA transfection (see above), after overexpression of a corresponding Tornado-vector-based construct (Supplementary Figure S5). HeLa cells were transfected with the Tornado-(CA)_100_ construct, and after three days, alternative splicing of *TJP1* and *BPTF/FALZ* was assayed by RT-PCR; in parallel, the effect on hnRNP L nuclear/cytoplasmic translocation was assessed by Western blot analysis (Supplementary Figure S5D; quantitated as described above). Clearly, both the hnRNP L sponging effect on alternative splicing as well as the hnRNP L translocation were reproduced, validating both effects under two very different experimental schemes, based on circRNA transfection (PIE system) or overexpression (Tornado system).

In conclusion, we have established here and validated a new concept of artificial circRNAs designed for specific protein sponge functions. As a paradigm we have used hnRNP L, a classical multidomain RNA-binding protein with multiple roles in RNA metabolism, in particular RNA processing. Two different types of hnRNP L sponges were designed, based on either CA-repeats [(CA)_20_ and (CA)_100_] or oligomerized CA-rich high-affinity binding motifs; either of them bound hnRNP L *in vitro* and *in vivo* with high efficiency, resulting in alternative splicing modulation comparable to RNAi knockdown effects. The high overexpression of an hnRNP L sponge also explains the dramatic translocation of the hnRNP L protein, from a predominant nuclear to cytoplasmic localization, where circRNAs accumulate. The strong correlation between circRNA-mediated sponging and classical siRNA-mediated RNAi effects on alternative splicing networks underlines that sponging effectively inactivates the RNA-binding protein (for direct comparisons of sponging versus RNAi, see Figures 4 and 5).

Therefore circRNA-mediated sponging of RNA-binding proteins should be considered as an alternative to RNAi-based knockdown. We were able to achieve similar effects on alternative splicing networks and the cellular distribution of an RNA-binding protein by either direct circRNA transfection or by overexpression. In sum, our results promise that designer circRNAs can be developed into a novel and highly specific new class of therapeutic RNAs, to be applied in cases where overexpressed (or mislocalized) RNA-binding proteins cause human disease, such as in many tumor tissues. For example, hnRNP L is overexpressed in prostate tumors, resulting in extensive changes of alternative splicing patterns, including those encoding prostate tumor-specific genes such as the androgen receptor (37).

## DATA AVAILABILITY

RNA-seq data were deposited in the Sequence Read Archive (PRJNA610182) of NCBI.

## Supplementary Material

gkaa1085_Supplemental_File
