# Supplementary material for: Design and application of circular RNAs with protein-sponge function
Source: Nucleic Acids Res. 2020 Nov 24;48(21):12326–35. doi: 10.1093/nar/gkaa1085 (PMC7708053; doi:10.1093/nar/gkaa1085)
Supplement: gkaa1085_Supplemental_File [file gkaa1085_supplemental_file.pdf]

Supplementary Information

Design and application of circular RNAs with protein-sponge function

Silke Schreiner[1], Anna Didio[1], Lee-Hsueh Hung[1], and Albrecht Bindereif[1]*

[1] Institute of Biochemistry, Justus-Liebig-University of Giessen, 35392 Giessen, Germany
* corresponding author, e-mail: albrecht.bindereif@chemie.bio.uni-giessen.de

Supplementary Materials and methods

HnRNP L binding to $(CA)_{100}$ RNA *in vitro*: mass-spectrometric analysis

10 μg biotinylated $(CA)_{100}$ circRNA (or control circRNA, both expressed by the PIE system and HPLC-purified, see below; for biotinylation, see main *Materials and Methods*) were bound to 300 μl MyOne Streptavidin C1 beads (binding capacity ~5,000 pmol/ml packed beads, Thermo Fisher Scientific), followed by addition of 300 μl pre-cleared nuclear extract [Cilbiotech, Belgium; diluted with 300 μl N100 buffer [50 mM Tris-Cl pH 8.0, 100 mM NaCl, 0.05% NP40 (v/v)], and incubation for one hour at 4°C. After washes (15 times from 100 mM up to 1000 mM NaCl), proteins were eluted from the beads by 50 μl SDS-loading buffer [100 mM Tris-Cl pH 6.8, 4% SDS (w/v), 0.2 bromphenol blue (w/v), 20% glycerol (v/v), and 200 mM ß-mercaptothanol] and run on a 4-20% Mini-Protean gel (Bio-Rad), followed by protein staining with colloidal Coomassie G250 (0.08% G250 Coomassie (w/v), 10% citric acid (w/v), 8% ammonium sulfate (w/v), and freshly added methanol (20% v/v), using peqGOLD pre-stained protein marker IV (VWR). Gel slices (CA1 to CA9 and K1 to K9, as indicated in **Supplementary Figure S1**) were subjected to trypsin digestion, followed by elution of the tryptic peptides with 1% trifluoric acid.

Matrix-assisted laser-desorption ionization time-of-flight mass spectrometry (MALDI-TOF-MS) was performed on an Ultraflex TOF/TOF mass spectrometer (Bruker Daltonics, Bremen) equipped with a nitrogen laser and a LIFT-MS/MS facility. The instrument was operated in the positive-ion reflectron mode using 2.5-dihydroxybenzoic acid and methylendiphosphonic acid as matrix. Sum spectra consisting of 200–400 single spectra were acquired. For data processing and instrument control the Compass 1.4 software package consisting of FlexControl 4.4, FlexAnalysis 3.4 4, Sequence Editor and BioTools 3.2 and ProteinScape 3.1. were used. External calibration was performed with a peptide standard (Bruker Daltonics).

Proteins were identified by MASCOT peptide mass fingerprint search (http://www.matrixscience.com) using the Uniprot Human database (version 20200226, 210438 sequence entries; $p<0.05$). For the search, a mass tolerance of 75 ppm was allowed, and oxidation of methionine as variable modification was used. As a result, as the major protein specifically bound by $(CA)_{100}$ circRNA, four hnRNP L isoforms were identified from the CA5 gel slice: A0A3B3ITJ4, M0QXS5, Q6NTA2, and B2R959 (each with sequence coverage between 33-35%).

HPLC purification of large circRNAs after PIE-mediated expression and circularization *in vitro*

Both the negative control and $(CA)_{100}$ circRNAs were generated by T7-transcription, followed by *in vitro* PIE-mediated circularization (30) and RNase R digestion [1 U RNase R (Lucigen)/µg RNA in RNase R buffer (20 mM Tris-Cl pH 8.0, 100 mM KCl, 0.1 mM $MgCl_2$); 20 min at 37°C]. CircRNAs were further purified by HPLC as following. 30 µg of RNA was heated at 80°C for 3 min and then placed on ice. RNA was loaded and run in RNase-free TE buffer (20 mM Tris-Cl pH 6.8, 1 mM EDTA) at a flow rate between 0.15 and 0.2 ml/min [4.6 x 300 mm size exclusion column SRT SEC-2000; particle size 5 µm; pore size 2000 Å (Sepax Technologies); ÄKTApurifier HPLC system (Amersham Biosciences)]. Individual circRNA-containing fractions were analyzed by E-gel electrophoresis [2% E-gel EX agarose gels (Invitrogen)], using high-

range RNA markers (Thermo Fisher Scientific). CircRNA-containing fractions were pooled and ethanol-precipitated.

Northern blot analysis of overexpressed circRNAs

For Northern blotting, total RNA was purified from HeLa cells transfected with circRNA overexpression constructs [$(CA)_{20}$, CA-SELEX X2, and CA-SELEX X4]. Aliquots were treated with RNase R [5 U RNase R (Lucigen)/µg RNA in RNase R buffer (20 mM Tris-Cl pH 8.0, 100 mM KCl, 0.1 mM $MgCl_2$); 60 min at 37°C], or left untreated. As a positive control for RNase R, a linear transcript (93 nts) with the same junction sequence was made by T7 transcription. 500 ng of total RNA samples or control RNA were separated by denaturing polyacrylamide/urea gel electrophoresis (10%), followed by transfer to a nylon membrane (semidry blotting), crosslinking by UV light, and probing with a single-stranded RNA probe (DIG RNA Labeling Mix; Roche) specific for the circular junction. For DNA-oligonucleotide sequences, see **Supplementary Table S2**. Probe detection with alkaline phosphatase-conjugated anti-DIG-Fab fragments and CDP-*Star* Chemiluminescence substrate were done as described in the Roche manual (NorthernMax hybridization buffer; Thermo Fisher Scientific). Low-range RNA size markers were from Thermo Fisher Scientific.

Legends to Supplementary Figures

Supplementary Figure S1.
Mass-spectrometric analysis of protein binding to $(CA)_{100}$ RNA *in vitro*.

Biotinylated $(CA)_{100}$ circRNA (or control circRNA) were bound to Streptavidin Dynabeads, followed by incubation with HeLa cell nuclear extract. After washing, bound protein was eluted and analyzed by SDS-PAGE and Coomassie staining (beads, 5 and 95%), together with aliquots of the input (1%) and the depleted supernatant (1%). Gel lanes for both $(CA)_{100}$ circRNA and control circRNA (95%) were sliced (CA1 to 9 and K1 to 9, respectively) and proteins identified by mass spectrometry (for details, see *Supplementary Materials and methods*). The red arrow indicates the major band identified in fraction CA5 as hnRNP L (below, the four UniProtKB accession numbers found, as well as details on molecular mass, number of peptides, and sequence coverage). *M*, protein markers (sizes in kDa).

Supplementary Figure S2.
HPLC purification of large circRNAs after PIE-mediated expression and circularization *in vitro*.

Both the negative control and $(CA)_{100}$ circRNAs (left and right panels) were generated by T7-transcription, followed by *in vitro* PIE-mediated circularization and RNase R digestion. CircRNAs were further purified by HPLC as described in *Supplementary Materials and methods*. Individual fractions were analyzed by E-gel electrophoresis (2% E-gel EX agarose gels), with the circRNA position marked (O), and using RNA markers (sizes in kb).

Supplementary Figure S3.
Absolute quantification of designer circRNA and cellular hnRNP L concentrations.

(A) Quantification of circRNA overexpression. The absolute cellular concentrations of overexpressed circRNAs were determined as described in *Materials and methods*, and summarized here as copy numbers per cell for PIE-expressed and transfected circRNAs [control, $(CA)_{100}$] as well as for circRNAs expressed by the Tornado-system [control, control/Broccoli, $(CA)_{100}$, $(CA)_{100}$/Broccoli, CA-SELEX X4, and CA-SELEX X4/Broccoli], with standard deviations (n=3).
(B) Quantification of cellular hnRNP L concentration in HeLa cell lysate. The hnRNP L concentration in HeLa cell lysate were determined by Western blotting with hnRNP L and GAPDH antibodies, comparing signals in lysate (1, 2.5, and 5 μl) and from purified recombinant GST-hnRNP L protein (5, 10, 30, and 50 ng), as described in *Materials and methods*. *M*, protein markers (sizes in kDa).

Supplementary Figure S4.

RNAi-mediated knockdown of hnRNP L (Western blot) and Northern blot analysis of overexpressed circRNAs.

(A) siRNA-mediated knockdown of hnRNP L: Western blot analysis. The siRNA-mediated knockdown of hnRNP L in HeLa cells was validated by Western blot, with control (ctr) versus hnRNP L-knockdown (ΔL), detecting hnRNP L and –as input control- GAPDH.

(B) Northern blot analysis of overexpressed circRNAs. Total RNA from HeLa cells transfected with circRNA overexpression constructs [$(CA)_{20}$, CA-SELEX X2, and CA-SELEX X4] was treated with RNase R or left untreated (-/+). As a positive control for RNase R, a linear transcript (93 nts) with the same junction sequence was used. RNA was analyzed by denaturing polyacrylamide/urea gel electrophoresis (10%), followed by Northern blotting, using a single-stranded DIG-labeled RNA probe specific for the circular junction. RNA markers (sizes in nts).

Supplementary Figure S5.

Overexpression of $(CA)_{100}$ circRNA: direct RNA analysis, alternative splicing modulation, and effect on nuclear-cytoplasmic distribution of hnRNP L.

(A) Schematic of $(CA)_{100}$ circRNA overexpression constructs (with and without Broccoli aptamer), based on the Tornado self-splicing system (reference 31; sizes of circRNAs given in parentheses).

(B) Direct RNA analysis of $(CA)_{100}$ and control circRNA overexpression in HeLa cells, two days post-transfection of $(CA)_{100}$, $(CA)_{100}$/Broccoli, as well as control- and control/Broccoli constructs. Total RNA (3 μg each) was analyzed by agarose electrophoresis (1.5%) and visualized by SYBR Gold (total RNA) and DFHBI (Broccoli staining). The red arrows mark overexpressed circRNAs (sizes in nts below). *M*, RNA markers (sizes in kb).

(C) Alternative splicing regulation of two known hnRNP L target genes. After a two-day overexpression in HeLa cells of $(CA)_{100}$ circRNA (or control circRNA; each with or without Broccoli), alternative splicing was assayed by RT-PCR for two known hnRNP L targets, *TJP1* and *BPTF/FALZ*, where hnRNP L functions as a splice repressor. The two RT-PCR products indicate exon inclusion (red arrows) and skipping, respectively; quantitation of exon inclusion (in %) is indicated in the respective lanes. *M*, DNA markers.

(D) Overexpression of $(CA)_{100}$ circRNA shifts nuclear-cytoplasmic distribution of hnRNP L. $(CA)_{100}$/Broccoli (or control/Broccoli) RNAs were overexpressed in HeLa cells, followed by cell fractionation after 72 hrs. Equivalent lysate amounts of total cells, cytoplasmic and nuclear fractions were analyzed by Western blotting for hnRNP L, GAPDH, and hnRNP A1. The distribution of hnRNP L between nuclear and cytoplasmic fractions was quantitated, based on Western signals (mean values and standard deviations given below the respective lanes; n=2).

Supplementary Figure S1

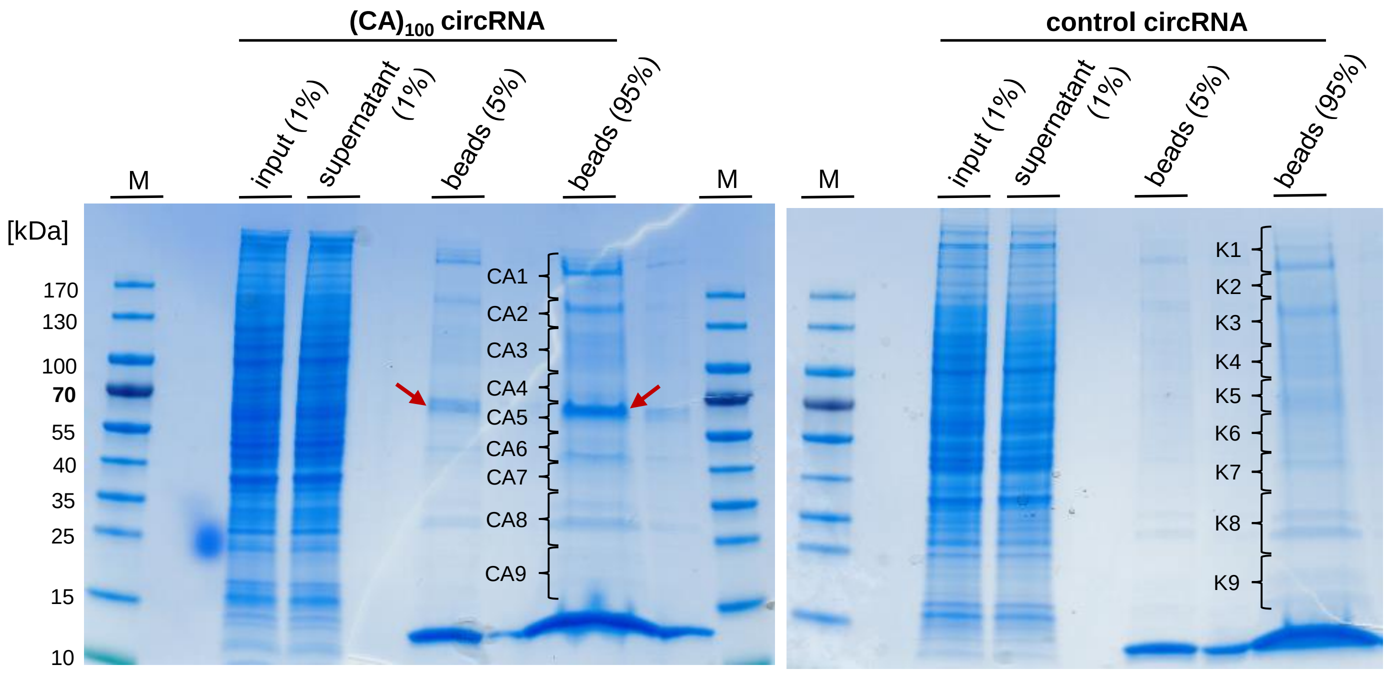

		molecular mass [kDa]	peptide #	sequence coverage [%]
**A0A3B3ITJ4**	Heterogeneous nuclear ribonucleoprotein L (fragment)	59.2	20	32.8
**M0QXS5**	Heterogeneous nuclear ribonucleoprotein L (fragment)	58.4	20	33.6
**Q6NTA2**	HNRNPL protein (fragment)	61.9	22	34.8
**B2R959**	cDNA, FLJ94229, highly similar to Homo sapiens heterogeneous nuclear ribonucleoprotein L (HNRPL)	60.2	22	34.6

Supplementary Figure S2

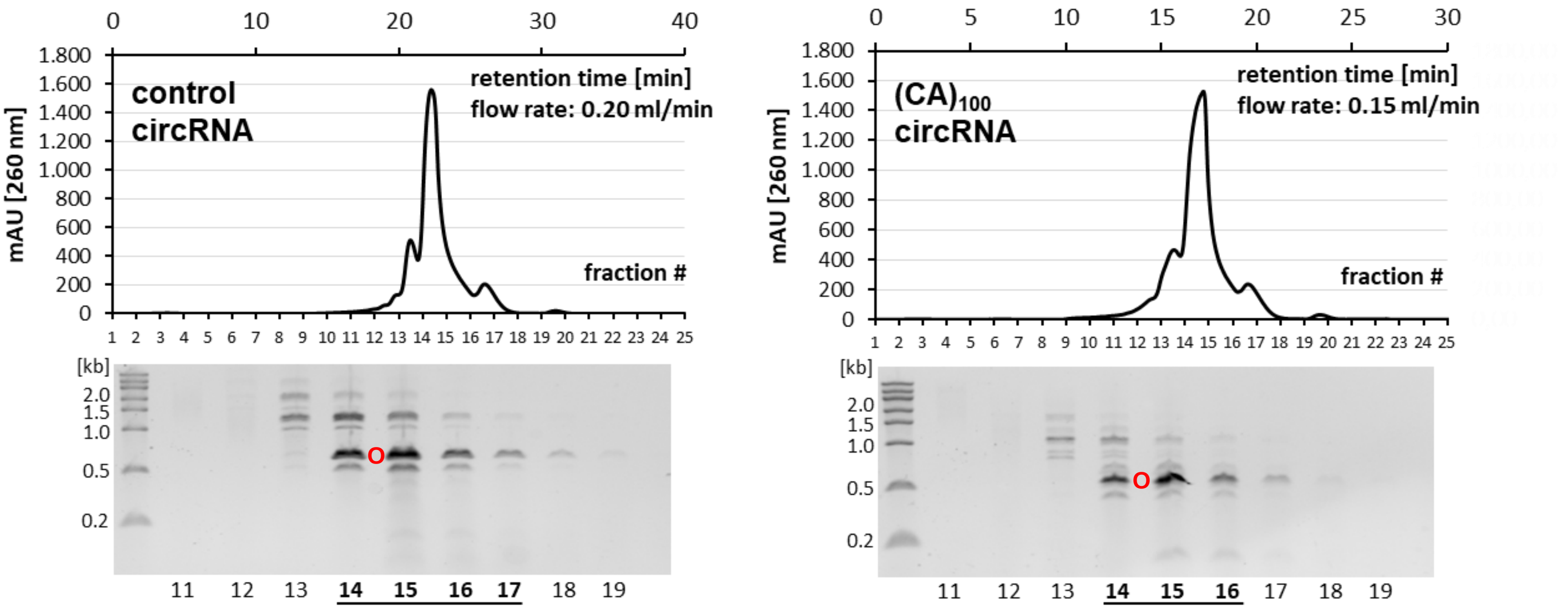

2% E-gel / Sybr-Gold

Supplementary Figure S3

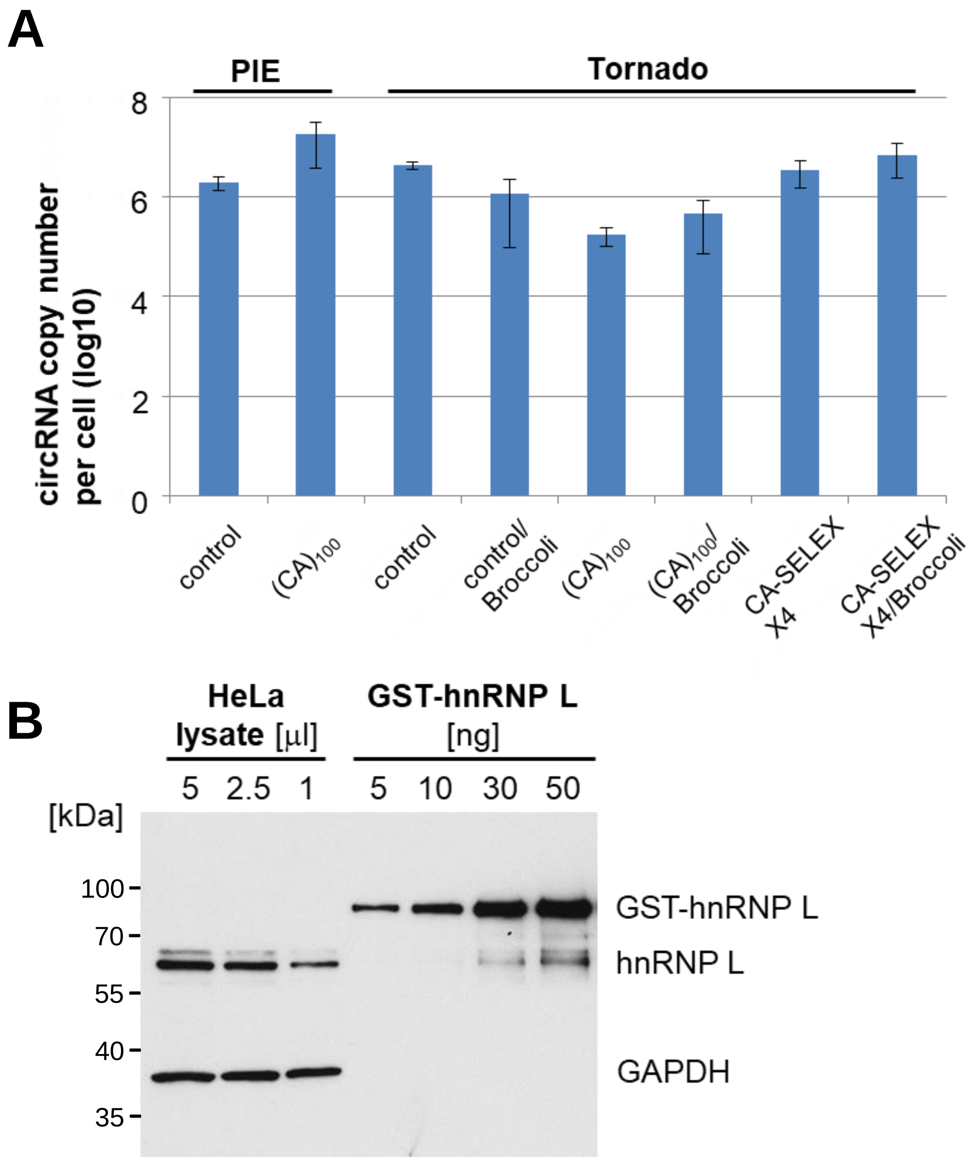

Supplementary Figure S4

A

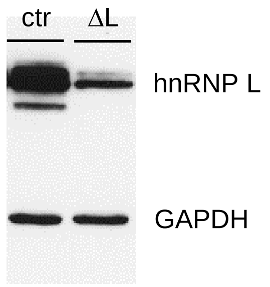

B

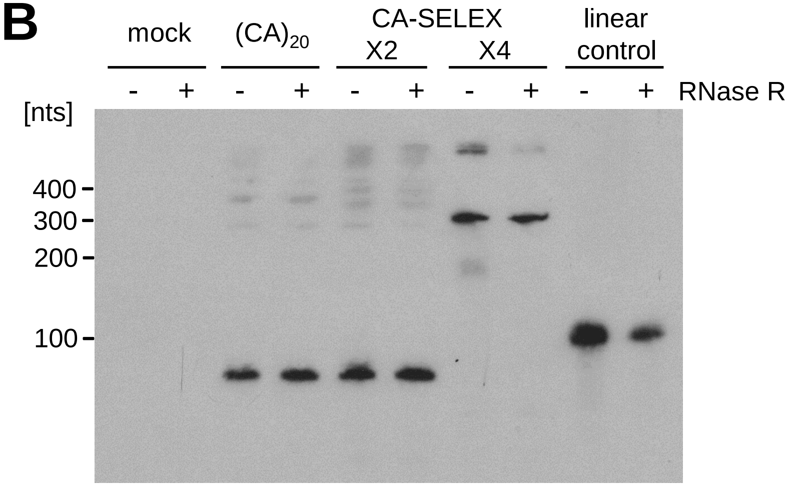

Northern blot

Supplementary Figure S5

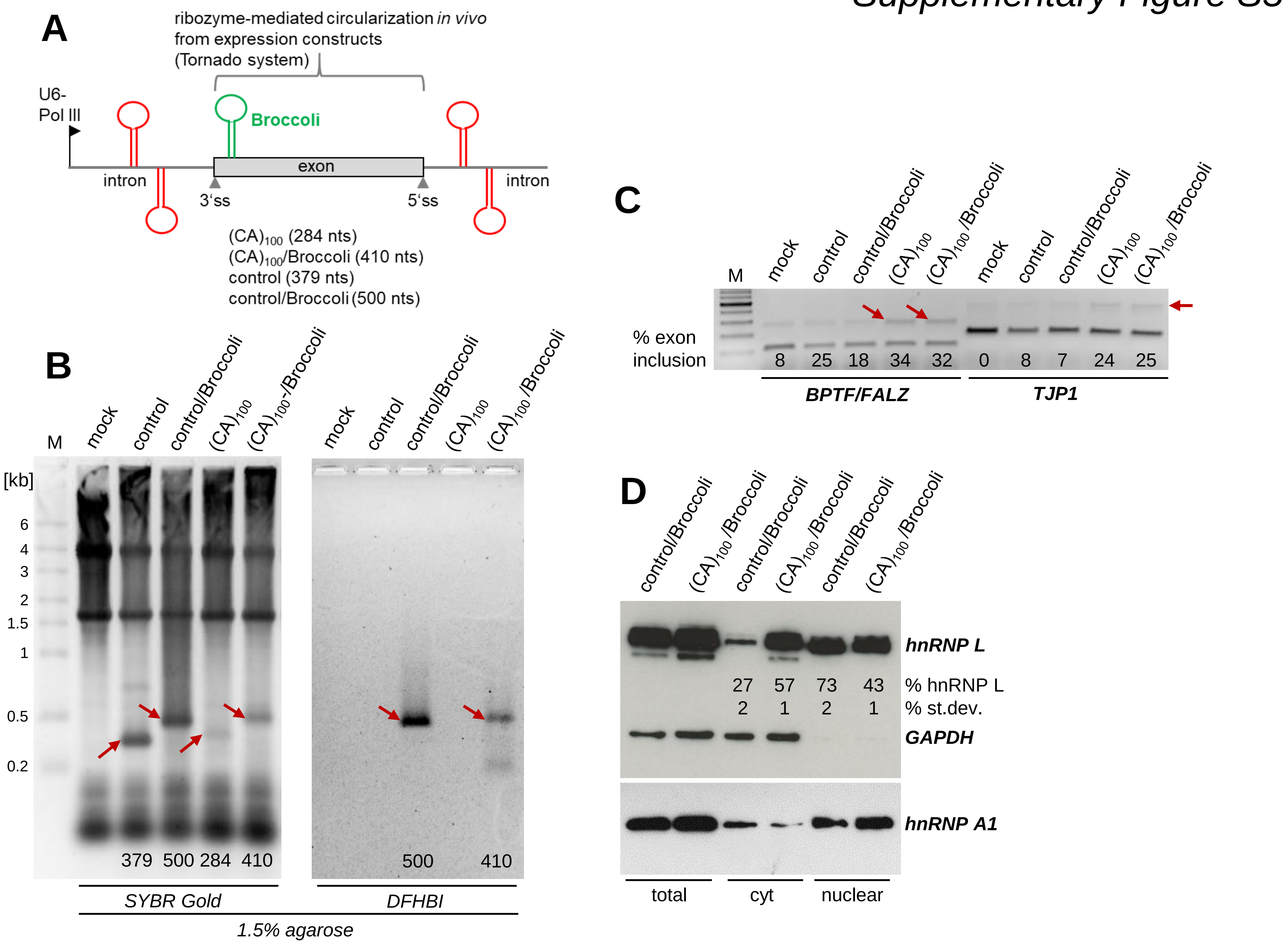

Supplementary Table 1A. Increased exon inclusion upon hnRNP L sponging

(**bold**: same effect also upon hnRNP L depletion by siRNA; **red**: RT-PCR validated)

gene_id	chromosome	strand	target exon position	skipping junction position
HP1BP3	chr1	-	21102217:21102498	21100103-21103090
CDC42BPA	chr1	-	227239582:227239686	227235711-227257478
LGALS8	chr1	+	236706215:236706340	236706087-236706860
LRP8	chr1	-	53723681:53723746	53723136-53723991
PARK7	chr1	+	8029145:8029208	8025485-8029405
CCDC18	chr1	+	93702819:93702937	93701947-93704867
RIF1	chr2	+	152325568:152325621	152325270-152326225
STAT1	chr2	-	191875093:191875175	191874730-191878251
PUM2	chr2	-	20478344:20478580	20463221-20482708
CBLB	chr3	-	105400323:105400454	105397415-105400568
WDR52	chr3	-	113127953:113128169	113125888-113135372
IQCB1	chr3	-	121507131:121507279	121500721-121508920
MBNL1	chr3	+	152173331:152173366	152165562-152174056
DLG1	chr3	-	196857673:196857849	196857542-196863413
WHSC1	chr4	+	1944066:1944292	1941505-1952799
OCIAD2	chr4	-	48901846:48901942	48899874-48906501
HNRNPD	chr4	-	83276048:83276154	83275307-83276457
C5orf42	chr5	-	37157415:37157522	37154095-37157772
GPBP1	chr5	+	56532940:56532999	56531859-56542127
CAST	chr5	+	96066484:96066564	96065429-96071876
LATS1	chr6	-	150018228:150018322	150016357-150022915
MDFIC	chr7	+	114654033:114654587	114619836-114655742
ANLN	chr7	+	36456689:36456799	36455493-36458853
SEMA3C	chr7	-	80546585:80546781	80546135-80548144
PCM1	chr8	+	17840742:17840798	17838264-17842956
HMBOX1	chr8	+	28903709:28903933	28902960-28904875
MYBL1	chr8	-	67478299:67478478	67477060-67478919
GAPVD1	chr9	+	128097451:128097531	128094908-128099297
GBA2	chr9	-	35743273:35743323	35741887-35744294
ADD3	chr10	+	111892063:111892158	111890244-111893084
LIN7C	chr11	-	27527864:27528002	27523467-27528263
C11orf82	chr11	+	82641199:82641316	82639980-82642774
WNK1	chr12	+	980431:980514	971436-987378
CARS2	chr13	-	111296412:111296529	111294868-111296732
IPO5	chr13	+	98660780:98660933	98660396-98662135
MAX	chr14	-	65568264:65568290	65560533-65569022
TJP1	chr15	-	30011981:30012220	30011342-30012562
KIF23	chr15	+	69733107:69733418	69732826-69737129
EDC3	chr15	-	74979432:74979520	74967483-74988221
BPTF	chr17	+	65925075:65925236	65924717-65925452
BPTF	chr17	+	65959449:65959622	65955991-65960328
DAZAP1	chr19	+	1419671:1420422	1418730-1421147
DDX27	chr20	+	47847560:47847652	47846886-47849843
ZFX	chrX	+	24193506:24193560	24191840-24197300
PRPF38B	chr1	+	109240569:109240639	109238959-109241226

MAGI3	chr1	+	114224834:114224924	114223958-114225519
DSTYK	chr1	-	205117333:205117467	205116873-205119808
MAP7D1	chr1	+	36640499:36640609	36639079-36641800
CRYZ	chr1	-	75196066:75196104	75190518-75198640
ODF2L	chr1	-	86851141:86851273	86850484-86852598
MAP4K4	chr2	+	102487956:102488147	102486877-102490109
FN1	chr2	-	216257654:216257926	216256537-216259251
AHSA2	chr2	+	61410682:61410797	61408540-61411847
SLC6A6	chr3	+	14509596:14509720	14509464-14513713
CAPN7	chr3	+	15282960:15283095	15282360-15283685
CRBN	chr3	-	3214190:3214330	3209477-3214460
SEMA3F	chr3	+	50212529:50212621	50211783-50214201
COL25A1	chr4	-	109774058:109774081	109773436-109780812
PPIP5K2	chr5	+	102523015:102523077	102522140-102526543
BOD1	chr5	-	173036581:173036673	173036437-173040134
CPEB4	chr5	+	173359453:173359503	173337607-173371970
C5orf34	chr5	-	43509247:43509477	43508768-43514908
SRD5A1	chr5	+	6653560:6653749	6652121-6656191
CAST	chr5	+	96062498:96062563	96058402-96063193
CAST	chr5	+	96064857:96064913	96063234-96065316
RWDD1	chr6	+	116895221:116895334	116892818-116901458
AHI1	chr6	-	135816952:135817036	135813429-135818326
SYNE1	chr6	-	152466622:152466690	152464900-152469180
PMPCB	chr7	+	102948043:102948155	102944937-102949399
INSIG1	chr7	+	155095535:155095605	155094556-155099951
MFSD3	chr8	+	145735743:145735890	145735398-145735981
CSPP1	chr8	+	68004038:68004118	67998345-68005778
DPY19L4	chr8	+	95746858:95746982	95738669-95750597
RC3H2	chr9	-	125620202:125620372	125618157-125620948
DPP7	chr9	-	140007197:140007257	140006682-140007405
BAG1	chr9	-	33262103:33262244	33261167-33262700
CEP78	chr9	+	80880775:80880822	80880459-80881358
FAM86C1	chr11	+	71502782:71502865	71500891-71507063
AKIP1	chr11	+	8934000:8934080	8933218-8936373
AMOTL1	chr11	+	94528177:94528326	94501726-94532556
HDAC7	chr12	-	48189689:48189799	48189550-48189990
CCDC77	chr12	+	518534:518587	514730-520913
IKBIP	chr12	-	99028074:99028191	99020544-99038301
TEX30	chr13	-	103419623:103419828	103418930-103420610
IPO5	chr13	+	98643835:98643950	98642791-98645146
TRAPPC6B	chr14	-	39623415:39623498	39621043-39627489
KTN1	chr14	+	56139890:56139973	56139730-56142553
SYNE2	chr14	+	64682004:64682072	64681188-64682966
MLH3	chr14	-	75506614:75506718	75505115-75508318
MYO9A	chr15	-	72311380:72311436	72302788-72313259
MORF4L1	chr15	+	79177309:79177425	79172921-79178483
SMG1	chr16	-	18911309:18911366	18908278-18937272
TNRC6A	chr16	+	24804794:24804970	24803138-24805865

PMM2	chr16	+	8904936:8905035	8900264-8905495
MPRIP	chr17	+	17083921:17083983	17083402-17088137
ALDH3A1	chr17	-	19642309:19642408	19641766-19642821
MED24	chr17	-	38191169:38191225	38189709-38191370
KIF1C	chr17	+	4902308:4902341	4901451-4903145
EXOC7	chr17	-	74086410:74086478	74085401-74087224
ENOSF1	chr18	-	677743:677872	677444-678696
TXNL4A	chr18	-	77746602:77746750	77737701-77748240
SSBP4	chr19	+	18542163:18542228	18541740-18542457
ZNF146	chr19	+	36716856:36716975	36709097-36719650
AXL	chr19	+	41745599:41745625	41745219-41748788
HNRNPUL1	chr19	+	41778522:41778659	41778140-41779887
THOC5	chr22	-	29927067:29927099	29925228-29927820
MORF4L2	chrX	-	102939609:102939657	102933579-102940099

Supplementary Table 1B. Increased exon skipping upon hnRNP L sponging

(**bold**: same effect also upon hnRNP L depletion by siRNA; **red**: RT-PCR validated)

gene_id	chromosome	strand	target exon position	skipping junction position
SCAMP3	chr1	-	155231448:155231525	155230450-155231877
EFHD2	chr1	+	15753646:15753780	15752514-15755089
ALPL	chr1	+	21900158:21900292	21896867-21902226
GALE	chr1	-	24125377:24125502	24125220-24125856
SYNC	chr1	-	33149611:33149690	33147461-33149859
MACF1	chr1	+	39844133:39844195	39838268-39844875
CRYZ	chr1	-	75172787:75172888	75172678-75175782
TYW3	chr1	+	75204374:75204472	75202306-75214435
Sep 15	chr1	-	87333736:87333785	87329288-87346345
CUL3	chr2	-	225422376:225422573	225400358-225449661
TEX261	chr2	-	71218960:71219113	71216946-71220826
LRCH3	chr3	+	197585705:197585776	197581316-197592294
GOLGA4	chr3	+	37327505:37327552	37323763-37330726
GOLGA4	chr3	+	37402734:37402796	37396678-37407571
QARS	chr3	-	49135425:49135550	49133512-49135625
CTBP1	chr4	-	1219148:1219354	1209993-1221987
CC2D2A (red)	chr4	+	15512870:15513046	15511863-15516330
RAPGEF2	chr4	+	160164926:160164943	160162520-160225494
LIMCH1	chr4	+	41640949:41640984	41621457-41646517
SLAIN2	chr4	+	48396593:48396670	48385801-48422142
PAM	chr5	+	102309820:102310140	102296933-102325976
FER	chr5	+	108290430:108290633	108281923-108294926
HMMR (red)	chr5	+	162894707:162894754	162891808-162896647
HMMR	chr5	+	162909648:162909794	162905806-162910026
FGFR1OP	chr6	+	167426996:167427055	167424381-167435897
SNX14	chr6	-	86248556:86248582	86246642-86251703
SLC7A2	chr8	+	17359766:17359811	17354746-17400827
ASPH	chr8	-	62546242:62546286	62538839-62550506
NCBP1	chr9	+	100407893:100408014	100407506-100409774
MELK	chr9	+	36657238:36657360	36651874-36665347
SMC3	chr10	+	112333465:112333503	112328771-112335094
ABI1	chr10	-	27044584:27044670	27040712-27047991
FAM208B	chr10	+	5766393:5766496	5765740-5768848
BICC1	chr10	+	60582986:60583064	60580228-60588521
PPP3CB (red)	chr10	-	75199630:75199659	75198178-75204483
MYOF	chr10	-	95152674:95152712	95148911-95155899
ARHGEF12	chr11	+	120280103:120280159	120278532-120291462
TEAD1	chr11	+	12900436:12900447	12886447-12901255
GANAB	chr11	-	62401782:62401847	62400986-62402293
SNHG1	chr11	-	62622360:62622410	62622023-62622605
TARBP2	chr12	+	53898919:53899046	53898599-53899433
SUPT20H	chr13	-	37622701:37622736	37622073-37625625
AKAP11 (red)	chr13	+	42871184:42871318	42869878-42872669
INF2	chr14	+	105181621:105181677	105181193-105185132
MTA1	chr14	+	105915696:105915746	105911848-105916395

CTAGE5	chr14	+	39790132:39790260	39788495-39796068
SOCS4	chr14	+	55498581:55498709	55494189-55509670
PAPOLA	chr14	+	97026986:97027048	97022750-97029156
SPNS1	chr16	+	28993222:28993377	28992936-28993677
ACACA	chr17	-	35567381:35567404	35564714-35578645
SKA2	chr17	-	57196680:57196856	57189706-57208642
PLSCR3	chr17	-	7296463:7296683	7296271-7296786
USF2	chr19	+	35760706:35760906	35760602-35761350
HNRNPM	chr19	+	8548042:8548095	8539128-8550487
RALY	chr20	+	32661625:32661672	32661441-32663680
FLNA	chrX	-	153585619:153585642	153583440-153585802
MED14	chrX	-	40525972:40526103	40523741-40531113
KDM6A	chrX	+	44919267:44919401	44918711-44919854
KDM6A	chrX	+	44921892:44921993	44920664-44922667
DIAPH2	chrX	+	96018099:96018119	96013257-96136578
SUCO	chr1	+	172546679:172546699	172544763-172546904
SAP130	chr2	-	128735559:128735663	128712918-128744359
CDCA7	chr2	+	174223983:174224219	174223565-174227954
ATF2	chr2	-	175986172:175986268	175983097-175994866
ABI2	chr2	+	204276008:204276094	204267457-204281631
FIP1L1	chr4	+	54306749:54306775	54294350-54308820
MATR3	chr5	+	138615624:138615747	138614818-138618428
DAB2	chr5	-	39388407:39388469	39383373-39388901
PAPD4	chr5	+	78952781:78952824	78945013-78964715
MAP7	chr6	-	136704809:136704919	136699006-136709531
JARID2	chr6	+	15410455:15410596	15374483-15452237
KIF13A	chr6	-	17790103:17790141	17788106-17794480
PNPLA8	chr7	-	108161920:108161965	108156018-108166473
HNRNPA2B1	chr7	-	26231042:26231958	26230748-26232115
ZMIZ2	chr7	+	44799750:44799827	44799059-44800024
EGFR	chr7	+	55214299:55214433	55211181-55218987
SHARPIN	chr8	-	145153984:145154108	145153897-145154180
REEP4	chr8	-	21996439:21996574	21996306-21996929
DPP7	chr9	-	140007813:140007948	140007739-140008317
ZFAND5	chr9	-	74978386:74978522	74975703-74979612
GDI2	chr10	-	5836848:5836982	5828013-5838726
JMJD1C	chr10	-	64979638:64979743	64977091-65024411
LTBP3	chr11	-	65307484:65307624	65307352-65307716
ERP29	chr12	+	112457560:112457698	112451413-112459954
ASUN	chr12	-	27067341:27067511	27067061-27068935
DNM1L	chr12	+	32891198:32891230	32890876-32892998
PEX5	chr12	+	7354837:7354947	7354437-7355208
DDHD1	chr14	-	53518562:53518645	53513667-53521156
DLGAP5	chr14	-	55615312:55615402	55615191-55617517
SNRPA1	chr15	-	101826419:101826498	101826006-101827113
SEC11A	chr15	-	85223944:85224063	85214013-85230856
NPRL3	chr16	-	167300:167374	162774-169125
AXIN1	chr16	-	341190:341297	339607-343488

KIFC3	chr16	-	57793037:57793065	57792821-57793640
SHMT1	chr17	-	18238873:18238989	18236602-18243357
LSM14A	chr19	+	34706029:34706205	34699956-34706501
ZC3H4	chr19	-	47572349:47572600	47571126-47575035
ASXL1	chr20	+	31017704:31017856	31017234-31019124
STX16	chr20	+	57234679:57234690	57227194-57242546
TTC3	chr21	+	38529463:38529516	38529208-38530433
RBFOX2	chr22	-	36152152:36152191	36142608-36155935
MST4	chrX	+	131203506:131203691	131202597-131205097

Supplementary Table 2. List of oligonucleotides.

**siRNA oligonucleotides**		
siRNA oligonucleotide	human hnRNPL 3´UTR 1581	5´-GACAUUUCUCUUUCCUUUATT-3´
siRNA oligonucleotide	luciferase GL2	5´-CGUACGCGGAAUACUUCGATT-3´

**RT-PCR primers for hnRNP L target detection**		
WHSC1	WHSC1 fwd	5´-TTCCACGGCAGCATCTTCAG-3´
	WHSC1 rev	5´-AGTCACTCCTCGCTCAGACT-3
C5orf42	C5orf42 fwd	5´-CAGTTCCATCGTCTGCAGAGT-3´
	C5orf42 rev	5´-CTTGCAGCCGTTTCCACAAT-3´
GPBP1	GPBP1 fwd	5´-TCCTGAGTATGAGAGAGAACCAA-3´
	GPBP1 rev	5´-TCTTAACTGGCTGTGACGGA-3´
CARS	CARS fwd	5´-CTGCTGTGTTTGGTGCCATC-3´
	CARS rev	5´-TCCACCACACCATGCAAGG-3´
RIF1	RIF1 fwd	5´-TCCATACCATGCCCAACAGA-3´
	RIF1 rev	5´-GAGTTGTCCCAGGCCTCTTG-3´
DLG1	DLG1 fwd	5´-GCATTGCTGGAGGTGTTGGA-3´
	DLG1 rev	5´-TGGTGATATCAGGTGGTGCA-3´
CDC42BPA	CDC42BPA fwd	5´-GCCTACCGATGCTCTGGATC-3´
	CDC42BPA rev	5´-AAGCCAGTTGAACCAGGACA-3´
LATS1	LATS1 fwd	5´-TGGGACGCATCATAAAGCCT-3´
	LATS1 rev	5´-TCTCGTCGAGGATCTTGGTAAC-3´
MYBL1	MYBL1 fwd	5´-ACCGCTTCTGGGAAGAAAGT-3´
	MYBL1 rev	5´-GGGAGTGGGGCATTTCATCA-3´
GBA2	GBA2 fwd	5´-GTTGGCAGCTTAACCCTGGA-3´
	GBA2 rev	5´-GGGACAGGACTTGCTGGTAC-3´
HMBOX	HMBOX fwd	5´-CCCAGATGAAGCAAAGAGGG-3´
	HMBOX rev	5´-TGGCTCTCCTCTTGATCTCCT-3´
LINC	LINC fwd	5´-TTAAGGGAGAAGATGGCGGC-3´
	LINC rev	5´-GCCTGAAGTTTCTGTGGTGG-3´
ZFX	ZFX fwd	5´-CCTCCCAGGCTCAAGTGATC-3´
	ZFX rev	5´-TGCTCAGGAATGATGACCGT-3´
CC2D2A	CC2D2A fwd	5´-CCAGGGAAAGAGGTAGAAAGGAC-3´
	CC2D2A rev	5´-GTCCTCGGCATCATCACCAT-3´
HMMR	HMMR fwd	5´-AGATACTACCTTGCCTGCTTCA-3´
	HMMR rev	5´-GCCTTGCTTCCATCTTTTCCA-3´
PPP3CB	PPP3CB fwd	5´-AGTGGAGTGTTAGCTGGAGGA-3´
	PPP3CB rev	5´-CCGAGGTGGCATTCTCTCAT-3´
AKAP11	AKAP11 fwd	5´-GCCAAGCATACTCCGGAAGA-3´
	AKAP11 rev	5´-TAGTTGGAATGGTCGGTGGG-3´
HNRNPM	HNRNPM fwd	5´-CCCTTTGGTGGTGGTATGGA-3´
	HNRNPM rev	5´-GACCCATCCTCTCGATCCCA-3´
TJP1	TJP1 fwd	5´-ATATCCTCCTTACTCACCACAAGC-3´
	TJP1 rev	5´-TTCAAAACATGGTTCTGCCTC-3´
FALZ	FALZ fwd	5´-TCATCAAACCTTTGCTACATGG-3´
	FALZ rev	5´-CTGACTGGTACCTGTACTTGATGG-3´

**DNA-oligonucleotides used for RT-(q)PCR**		
ß-actin	ß-actin fwd	5´-TGGACTTCGAGCAAGAGATG-3´
	ß-actin rev	5´-GTGATCTCCTTCTGCATCCTG-3´
snoRD	snoRD U78 fwd	5´-GTGTAATGATGTTGATCAAATGT-3´
	snoRD U78 rev	5´-TTCTTCAGTGTTACCTTGTCTA-3´
GAPDH	GAPDH fwd	5´-GAGTCAACGGATTTGTGCGT-3´
	GAPDH rev	5´-GATCTCGCTCCTGGAAGATG-3´
BGH	Bgh rev	5´-TAGAAGGCACAGTCGAGG-3´
$(CA)_{100}$ circ (Wilusz)	$(CA)_{100}$ circ fwd	5´-ACACACACACACAGATCCACT-3´
	$(CA)_{100}$ circ rev	5´-GTGTGTGTGTGGGATCCGAG-3´
PIE (CA)100 circ	$(CA)_{100}$ circ fwd	5´-CACACACACAAGATCCACTA -3´
	$(CA)_{100}$ circ rev	5´-TGTGTGTGTGTGGATCCTCT-3´
PIE intron	PIE intron fwd	5´-ATCGGAAGGTGCAGAGACTC-3´

Tornado (CA)$_{100}$ circ	Tornado circ (CA)$_{100}$ fwd	5´-CACAAGATCCACTAGTAACGG-3´
	Tornado circ (CA)$_{100}$ rev	5´-TGTGGGATCCGAGCGGGA-3´
Tornado (CA)$_{100}$ circ	Tornado circ (CA)$_{100}$ SacII fwd	5´-CACAAGATCCACTAGTCCGCG-3´
	Tornado circ (CA)$_{100}$ NotI rev	5´-TGTGGGATCCGAGCGCGGC-3´
Tornado (CA)$_{20}$ circ	Tornado (CA)$_{20}$ circ fwd	5´-TCGGCGTGGACTGTAGAAC-3´
	Tornado (CA)$_{20}$ circ rev	5´-TGTGTGTGTGTGTGGCGG-3´
Tornado CA-SELEX X2 circ	Tornado CA-SELEX X2 circ fwd	5´-TCGGCGTGGACTGTAGAAC-3´
	Tornado CA-SELEX X2 circ rev	5´-CATGTATGCGGCCGCACT-3´
Tornado CA-SELEX X4 circ	Tornado CA-SELEX X4 circ fwd	5´-CAAGATGTGGCCGCGGTC-3´
	Tornado CA-SELEX X4 circ rev	5´-ATGTATGGATCCGCGGCCG-3´
Tornado CA-SELEX X4 Broccoli circ	Tornado CA-SELEX X4 Broccoli circ fwd	5´-GGTCGGCGTGGACTGTAG-3´
	Tornado CA-SELEX X4 Broccoli circ rev	5´-GATACGAATATCTGGACCCGACCGTC-3´
Tornado Broccoli circ	Tornado Broccoli circ fwd	5´-GGTCGGCGTGGACTGTAG-3´
	Tornado Broccoli circ rev	5´-GATACGAATATCTGGACCCGACCGTC-3´
Control circ	Control circ fwd	5´-AGGATGAGGATCGTTTCGCA-3´
	Control circ rev	5´-CGGACTGGCTTTCTACGTGT-3´
Precursor Wilusz	Precursor Wilusz fwd	5´-CTGATAGTGCCTCGATGTGC-3´

**DNA-oligonucleotides used for *in vitro* transcription and biotin pull-down**		
T7 (CA)$_{10}$	T7 (CA)$_{10}$ fwd	5´-TAATACGACTCACTATAGGGAGTAAGCCACACACACACACACACACAGCTTACAGTA-3´
	T7 (CA)$_{10}$ rev	5´-TACTGTAAGCTGTGTGTGTGTGTGTGTGTGGCTTACTCCCTATAGTGAGTCGTATTA-3´
T7 (CA)$_{15}$	T7 (CA)$_{15}$ fwd	5´-TAATACGACTCACTATAGGGAGTAAGCCACACACACACACACACACACACACACACAGCTTACAGTA-3´
	T7 (CA)$_{15}$ rev	5´-TACTGTAAGCTGTGTGTGTGTGTGTGTGTGTGTGTGTGTGGCTTACTCCCTATAGTGAGTCGTATTA-3´
T7 (CA)$_{20}$	T7 (CA)$_{20}$ fwd	5´-TAATACGACTCACTATAGGGAGTAAGCCACACACACACACACACACACACACACACACACACACACACAGCTTACAGTA-3´
	T7 (CA)$_{20}$ rev	5´-TACTGTAAGCTGTGTGTGTGTGTGTGTGTGTGTGTGTGTGTGTGTGTGTGTGGCTTACTCCCTATAGTGAGTCGTATTA-3´
T7 L#51 40nt	T7 L#51 40nt fwd	5´-TAATACGACTCACTATAGGGAGTAAGCATACATGACACACACACGCAGCTTACAGTA-3´
	T7 L#51 40nt rev	5´-TACTGTAAGCTGCGTGTGTGTGTCATGTATGCTTACTCCCTATAGTGAGTCGTATTA-3´
T7 OR 40 (control)	T7 OR 40 fwd (control)	5´-TAATACGACTCACTATAGGGAGTAAGCCCTGCCTGTCTATTGATGTCGCTTACAGTA-3´
	T7 OR 40 rev (control)	5´-TACTGTAAGCGACATCAATAGACAGGCAGGGCTTACTCCCTATAGTGAGTCGTATTA-3´

**DNA-oligonucleotides used for *in vitro* transcription of Tornado absolute quantification standards (qPCR standards)**		
T7 OR CA-SELEX X4	T7 OR CA-SELEX X4 fwd	5´-TAATACGACTCACTATAGGGAGTAAGCAACCATGCCGAGTGCGGCCGCGGATCCATACATGCAAGATGTGGCCGCGGTCGGCGTGGACTGTAGGCTTACAGTA-3´
	T7 OR CA-SELEX X4 rev	5´-TACTGTAAGCCTACAGTCCACGCCGACCGCGGCCACATCTTGCATGTATGGATCCGCGGCCGCACTCGGCATGGTTGCTTACTCCCTATAGTGAGTCGTATTA-3´
T7 OR CA-SELEX X4 Broccoli	T7 OR CA-SELEX X4 Broccoli fwd	5´-TAATACGACTCACTATAGGGAGTAAGCAACCATGCCGAGTGCGGCCGCTTGCCCGGAGACGGTCGGGTCCAGATATTCGTATCTGTGGCCGCGGTCGGCGTGGACTGTAGGCTTACAGTA-3´
	T7 OR CA-SELEX X4 Broccoli rev	5´-TACTGTAAGCCTACAGTCCACGCCGACCGCGGCCACAGATACGAATATCTGGACCCGACCGTCTCCGGGCAAGCGGCCGCACTCGGCATGGTTGCTTACTCCCTATAGTGAGTCGTATTA-3´

**DNA-oligonucleotides used for cloning in PIE Vector**		
PIE control	PIE control BamHI fwd	5´-GTGGATCCTCCGGAGGAAGCGGAACACG-3´
	PIE control XhoI rev	5´-GTCTCGAGAGCACACTGGAGACGTAATC-3´
PIE (CA)$_{100}$	PIE (CA)$_{100}$ BamHI fwd	5´-TCCGGGACGACTCGGATCCCACA-3´
	PIE (CA)$_{100}$ XhoI rev	5´-GTCTCGAGCCGTTACTAGTGGATCTTGTG-3´

**DNA-oligonucleotides used for deletion of BGH signal**		
BGH	Bgh intern fwd	5´-CCACTCCCACTGTCCTTTCC-3´
	Bgh intern rev	5´-TGCCTGCTATTGTCTTCCCA-3´

**DNA-oligonucleotides for cloning of Tornado constructs**		
(CA)$_{20}$	NotI_(CA)$_{20}$_SacII fwd	5´-GGCCGCCACACACACACACACACACACACACACACACACACACACAGT-3´
	NotI_(CA)$_{20}$_SacII rev	5´-GGCCACTGTGTGTGTGTGTGTGTGTGTGTGTGTGTGTGTGTGTGTGGC-3´
SELEX X2	NotI_SELEX X2_SacII fwd	5´-GGCCGCATACATGACACACACACGCAATACATGACACACACACACGCAGTCGCCGC-3´
	NotI_SELEX X2_SacII rev	5´-GGCCACTGCGTGTGTGTGTCATGTATTGCGTGTGTGTGTCATGTATGC-3´
SELEX X4	NotI_SELEX X4_SacII fwd	5´-GGCCGCGGATCCATACATGACACACACACGCAATATATACATGACACACACACGCAATATATACATGACACACACACGCAATATATACATGACACACACACGCAAGATGTGGCCGC-3´
	NotI_SELEX X4_SacII rev	5´-GGCCACATCTTGCGTGTGTGTGTCATGTATATATTGCGTGTGTGTGTCATGTATATATTGCGTGTGTGTGTCATGTATATATTGCGTGTGTGTGTCATGTATGGATCCGC-3´
SELEX X4	KflI_SELEX X4_KflI fwd	5´-ATGATGGGTCCCAGAGGATCCATACATGACACACACACGCAATATATACATGACACACACACGCAATATATACATGACACACACACGCAATATATACATGACACACACACGCAAGATCTCGAGAAGGGTCCCATCATT-3´
	KflI_SELEX X4_KflI rev	5´-AATGATGGGACCCTTCTCGAGATCTTGCGTGTGTGTGTCATGTATATATTGCGTGTGTGTGTCATGTATATATTGCGTGTGTGTGTCATGTATATATTGCGTGTGTGTGTCATGTATGGATCCTCTGGGACCCATCAT-3´
Tornado (CA)$_{100}$	Tornado (CA)$_{100}$ NotI fwd	5´-TAAGCAGCGGCCGCGCTCGGATCCCACA-3´
	Tornado (CA)$_{100}$ SacII rev	5´-TGCTTACCGCGGACTAGTGGATCTTGTG-3´

Tornado $(CA)_{100}$	Tornado $(CA)_{100}$ KflI fwd	5´-TAAGCAGGGTCCCGCTCGGATCCCACA-3´
	Tornado $(CA)_{100}$ KflI rev	5´-TGCTTACCGCGGACTAGTGGATCTTGTG-3´
Tornado control	Tornado control NotI fwd	5´-TAAGCAGCGGCCGCTCCGGAGGAAGCGGAA-3´
	Tornado control SacII rev	5´-TGCTTACCGCGGAGCACACTGGAGACGT-3´
Tornado control	Tornado control KflI fwd	5´-TAAGCAGGGTCCCTCCGGAGGAAGCGGAA-3´
	Tornado control KflI rev	5´-TGCTTAGGGTCCCAGCACACTGGAGACGT-3´

**DNA-oligonucleotides for Northern blot (Supplementary Figure S4B)**		
T7 circular-junction probe	T7 circ-junction fwd	5´-TAATACGACTCACTATAGGGTTGCGGCCGCACTCGGCATGGTTCTACAGTCCACGCCGACCGCGGTTTTTTTTTTTTTTTTTTTT-3´
	T7 circ-junction rev	5´-AAAAAAAAAAAAAAAAAAAACCGCGGTCGGCGTGGACTGTAGAACCATGCCGAGTGCGGCCGCAACCCTATAGTGAGTCGTATTA-3´
T7 Tornado standard	T7 Tornado standard fwd	5´-TAATACGACTCACTATAGGGTCGGTGCGCGCCCCCTACAAAAACCGCGGTCGGCGTGGACTGTAGAACCATGCCGAGTGCGGCCGCAACAGCCAAGCTCGCCGCGGTGCC-3´
	T7 Tornado standard rev	5´-GGCACCGCGGCGAGCTTGGCTGTTGCGGCCGCACTCGGCATGGTTCTACAGTCCACGCCGACCGCGGTTTTTGTAGGGGGCGCGCACCGACCCTATAGTGAGTCGTATTA-3´
